# Screening of Bacteriophage Encoded Toxic Proteins with a Next Generation Sequencing-Based Assay

**DOI:** 10.3390/v13050750

**Published:** 2021-04-24

**Authors:** Jutta Kasurinen, Cindy M. Spruit, Anu Wicklund, Maria I. Pajunen, Mikael Skurnik

**Affiliations:** 1 Department of Bacteriology and Immunology, Medicum, Human Microbiome Research Program, Faculty of Medicine, University of Helsinki, 00290 Helsinki, Finland; jutta.kasurinen@helsinki.fi (J.K.); c.m.spruit@uu.nl (C.M.S.); anumaria.wicklund@gmail.com (A.W.); maria.pajunen@helsinki.fi (M.I.P.); 2 Division of Clinical Microbiology, HUSLAB, University of Helsinki and Helsinki University Hospital, 00290 Helsinki, Finland

**Keywords:** bacteriophage, hypothetical proteins of unknown function, next-generation sequencing, toxic protein screen

## Abstract

Bacteriophage vB_EcoM_fHy-Eco03 (fHy-Eco03 for short) was isolated from a sewage sample based on its ability to infect an *Escherichia coli* clinical blood culture isolate. Altogether, 32 genes encoding hypothetical proteins of unknown function (HPUFs) were identified from the genomic sequence of fHy-Eco03. The HPUFs were screened for toxic properties (toxHPUFs) with a novel, Next Generation Sequencing (NGS)-based approach. This approach identifies toxHPUF-encoding genes through comparison of gene-specific read coverages in DNA from pooled ligation mixtures before electroporation and pooled transformants after electroporation. The performance and reliability of the NGS screening assay was compared with a plating efficiency-based method, and both methods identified the fHy-Eco03 gene *g05* product as toxic. While the outcomes of the two screenings were highly similar, the NGS screening assay outperformed the plating efficiency assay in both reliability and efficiency. The NGS screening assay can be used as a high throughput method in the search for new phage-inspired antimicrobial molecules.

## 1. Introduction

The emergence of bacteria resistant to all currently available antibiotics is, and has long been, managed by modifying existing drugs to re-gain effectiveness against resistant bacteria [1]. Drug development, however, cannot keep up with bacterial evolution, as the environment is under a constant load of antimicrobials, causing the persistence and spread of resistance genes [2]. The development of new antibiotic classes is expensive and often too futile, as a majority of promising candidates do not make it through the clinical testing processes. Therefore, new sources for antimicrobial drugs are in high demand [3], such as lytic proteins produced by bacteriophages during the infection cycle that include virion-associated peptidoglycan hydrolases and endolysins in conjunction with holins that have already been used to some extent as antibacterials [4,5].

Phage genomes also encode numerous proteins for which the functions and structures are still unknown. These proteins are annotated in the sequence databases as “hypothetical proteins with unknown function” (HPUFs). Based on previous studies, many HPUFs include small molecules with toxic properties (toxHPUFs) that phages utilize during the infection cycle to hamper the cellular functions and defense mechanisms of the bacterial host [6]. Traditionally, toxic gene products have been detected by the inefficient transformation of bacterial cells with a plasmid carrying the toxin-encoding gene [6,7,8,9,10,11]. In the plating efficiency assay, the number of transformants obtained with the HPUF-encoding genes is compared to the number of transformants obtained with control genes encoding known toxic and non-toxic proteins. In practice, the PCR-amplified HPUF genes are ligated into a plasmid vector and electroporated individually into host bacteria; the resulting transformants are enumerated. This approach includes several difficult-to-standardize steps that all cause day-to-day, experiment-to-experiment, and batch-to-batch variation, even when the toxic and non-toxic controls are included in every batch. The reproducibility can be improved by increasing the number of replicates; however, such an approach does not allow high throughput without robotics.

In the next-generation-sequencing (NGS)-based assay described in this study, most of these issues are avoided by transforming all the selected genes simultaneously as a pooled ligation mixture. Using NGS read coverages, the relative abundance of correctly ligated inserts can be determined for a pooled ligation mixture and for the plasmids isolated from the pooled transformants. We describe and validate here the NGS screening assay by carrying out screening with five known toxic and four non-toxic genes of phages fHe-Kpn01 [10], ϕR1-RT [8], and T4 [12] and 32 HPUF-encoding genes of *Escherichia* phage fHy-Eco03 in a comparative study with the plating assay [10].

## 2. Materials and Methods

### 2.1. Bacterial Strains, Bacteriophages and Culture Media

The bacteriophages and bacterial strains used in this study are listed in Table 1 and Table S1. Plasmid pU11L4 used for cloning in this study was isolated from *E. coli* strain DH10B/pU11L4. The electrocompetent DH10B and DH5α cells were prepared as described previously [13]. All bacterial strains used in this study were grown on LB agar (1.5%, *w/v*) or in LB broth supplemented with 100 µg/mL ampicillin (Sigma-Aldrich, St. Louis, MO, USA) unless mentioned otherwise, at 35 °C or 37 °C. Transformed *E. coli* DH10B cells were grown in SOC medium (0.5% yeast extract, 2% tryptone, 10 mM NaCl, 2.5 mM KCl, 10 mM MgCl_2_, 10 mM MgSO_4_, 20 mM glucose pH 7.0) and transformed DH5α cells were grown on M9t minimal medium (2.2 mM KH_2_PO_4_, 3.4 mM Na_2_HPO_4_, 0.94 mM NH_4_Cl, 0.86 mM NaCl, 0.2% (*w/v*) tryptone, 2.0 mM MgSO_4_, 0.10 mM CaCl_2_ and 3.0 × 10^−3^ mM vitamin B1).

### 2.2. Phage Isolation and Purification

fHy-Eco03 was isolated from a municipal sewage sample collected in Hyvinkää, Finland, using clinical *E. coli* strain #5509 (Table 1 and Table S1) as the host. Strain #5509 was used to propagate the phage. Phage lysates were produced from semiconfluent soft-agar plates as described elsewhere [17]. The phage lysates were then concentrated, purified using ultracentrifugation with a glycerol step gradient [18], and stored at 4 °C as described earlier [10].

### 2.3. Electron Microscopy

Phage particles were sedimented by centrifugation for 2 h (16,000× *g* at 4 °C) and resuspended in 0.1 M ammonium acetate (pH 7.2). Afterward, 200 mesh Formvar-coated copper grids were used to allow the phage particles to sediment for 1 min. Negative staining was done using 1% uranyl acetate at pH 4.2 (method modified from [19]). A JEOL JEM1400 electron microscope, operated at 80 kV, and an Olympus Morada CCD camera were used to image the phage particles (Department of Virology, University of Helsinki, Helsinki, Finland).

### 2.4. Host Range Determination

The host range of fHy-Eco03 on 50 *E. coli* strains (Table S1) was determined by pipetting 10 μL droplets of serial dilutions of concentrated phage stocks on lawns of different bacterial strains prepared on LB agar plates, and the plates were incubated until the next day at 37 °C. The double-layer method was used to confirm positive droplet test results using phage preparations with appropriate dilutions.

Infection growth curves on phage-sensitive *E. coli* strains were performed as follows. An overnight bacterial culture was diluted 500-fold in fresh LB medium, and 180 μL aliquots were distributed into Bioscreen Honeycomb 2 plates wells (Growth Curves Ab Ltd., Helsinki, Finland), where they were mixed with 20 μL of different fHy-Eco03 phage stock dilutions. The phage stock and bacterial culture were mixed to achieve multiplicity of infection (MOI) values ranging between 0.5 and 500. The controls consisted of 180 μL of bacterial culture and 20 μL of fresh LB medium, or just 200 μL of fresh LB medium alone. The growth experiment was carried out at 37 °C using a Bioscreen C incubator (Growth Curves Ab Ltd., Helsinki, Finland) with continuous shaking. The OD_600_ of the cultures was measured every 45 min for up to 15 h. The averages were calculated from values obtained for the bacteria grown in, at minimum, triplicate wells.

### 2.5. Genome Sequencing and Analysis

Phage DNA was obtained from high-titer phage preparations as described earlier [17], and sequenced at Eurofins Genomics. The next-generation sequencing DNA library (insert size of 625 ± 311) was paired-end sequenced using Illumina MiSeq sequencer (Illumina, San Diego, CA, USA) with a read length of 150 nucleotides. The A5-miseq integrated pipeline for de novo assembly of microbial genomes was used to assemble the genome sequence [20]. The termini of the phage genome were identified using PhageTerm [21], and confirmed by restriction digestions and comparisons to related phages. The orientation of the genome was arranged similarly to the sequences of closely related homologs as found in a nucleotide BLAST search. The genes were annotated with RAST software [22] and validated manually, confirming also that the predicted genes were accompanied by a properly located ribosomal binding site. The Geneious Prime v 11.1.5 [23] was used for visualization of the phage genome.

A protein BLAST against the non-redundant protein sequences database (release update from 6 February 2021) was performed for every predicted gene product and the two results with the lowest E-values were recorded (Table S2). Furthermore, every gene product was analyzed using HHpred [24], and the best hits with a probability above 50% and an E-value below 1 were recorded (Table S2). The presence of tRNAs was investigated using tRNAscan-SE [25]. In addition, ResFinder 3.1 [26] and VirulenceFinder 2.0 [27] software were used. Phylogenetic trees of complete phage genomes at the nucleotide level were constructed using VICTOR [28]. The complete genome sequence with annotation was deposited in the NCBI nucleotide database (GenBank) under accession number MW602648.

### 2.6. Proteomics

The protein content of purified phages (as tryptic peptides) was analysed using liquid chromatography–tandem mass spectrometry (LC-MS/MS) at the Proteomics Unit, Institute of Biotechnology, University of Helsinki as described earlier [8,29]. Calibrated tryptic peptide peaks were searched against the predicted tryptic peptides from the amino acid sequences of all, even the non-annotated, open reading frames (ORFs) in the genome of fHy-Eco03. The proteins identified by LC-MS/MS analysis as having two or more unique tryptic peptides and over 5% sequence coverage were annotated as phage (structural) proteins.

### 2.7. DNA Methods

Restriction digestions of purified fHy-Eco03 DNA were performed with restriction endonucleases *Nco*I, *Not*I, *Sca*I, *Sph*I (Thermo Fisher Scientific, Waltham, MA, USA), *Afl*II, *Eag*I, *Sau*3AI, and *Sma*I (New England Biolabs, Ipswich, MA, USA) in appropriate digestion buffers.

All plasmid isolations were performed with a commercial Nucleobond Xtra Midi kit (MACHEREY-NAGEL, Düren, Germany) according to the protocol for high-copy number plasmids. Plasmid pU11L4 was double digested with restriction enzymes *Not*I and *Nco*I, or with *Nhe*I and *Not*I (Thermo Fisher Scientific, USA), if an internal *NcoI* site was present in the sequence of the gene in question (Tables S3 and S4). Toxic and non-toxic control genes (Table S3) and the HPUF-encoding (Table S4) genes of phage fHy-Eco03 were cloned as PCR-fragments into the multiple cloning site of plasmid pU11L4 (Figure S1) with a three-molar excess of the insert.

The recombinant plasmids were transformed to electrocompetent *E. coli* DH10B cells, having a transformation efficiency of approximately 10^9^ CFU per µg of intact pU11L4 plasmid. The electroporation was performed in 0.2 mm cuvettes (Bulldog Bio, Portsmouth, NH, USA) by combining approximately 10 ng of the recombinant vector to 45 µL of electrocompetent DH10B cells. The pulse was given with a Gene Pulser™ apparatus (Bio-Rad Laboratories, Hercules, CA, USA), with the settings of 200 Ω, 25 uF and 2.5 kV. Transformed cells were suspended immediately in 950 µL of SOC medium and incubated at 35 °C for 1 h in slow rotation; afterwards, 50 µL was pipetted on LB ampicillin plates. Plates were incubated overnight at 37 °C. Transformations of the HPUF-encoding genes of fHy-Eco03 were done in batches of 4 to 6 genes with the *g178* gene of phage φR1-RT as a non-toxic control in each batch [8,10]. The relative CFUs were determined from triplicate platings of two biological replications per gene.

For the NGS screening assay, the individual fHy-Eco03 HPUF-encoding gene ligations were combined to pooled samples in two batches, containing all the HPUF-encoding genes, without any control genes. The pooled ligation mix samples were purified and concentrated with NucleoSpin^®^ Gel and a PCR Clean-up kit (MACHEREY-NAGEL, Germany) according to instructions, and eluted in 20 µL; 1 µL was used for electroporation as described above. After the initial 1 hr incubation in SOC, the whole suspension was spread in 50 µL aliquots on twenty LB ampicillin plates and grown overnight at 37 °C. All the obtained transformant colonies were pooled together, and the pool was grown aerated to the late logarithmic phase in ampicillin supplemented SOC (3 h at 37 °C). Plasmid isolation from the culture was carried out as described above using the Nucleobond Xtra Midi kit.

### 2.8. Bioinformatics

**Figure 1:**
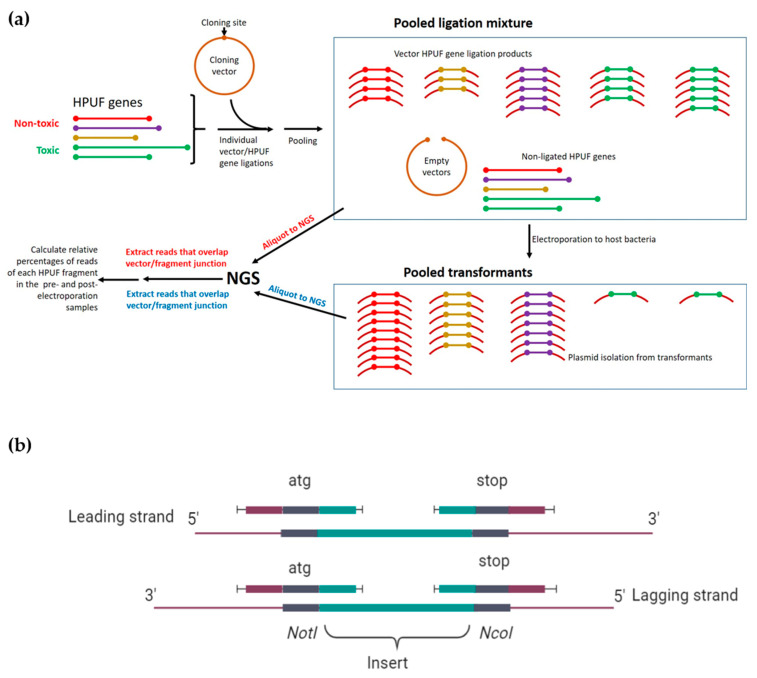
Overview of the NGS-based screening approach. (**a**) Illustration of the NGS-based screening assay approach to identify phage encoded toxHPUFs. (**b**) Schematic illustration of the four in silico generated DNA sequences over the ligation joints used in the extraction of the sequence reads from the total sequence read files. The image in (**b**) was created in BioRender.com.

The NGS-based screening approach is outlined in Figure 1. The ligation mixture and transformant samples containing plasmid DNA were sequenced with the 150 bp paired-end protocol in the Illumina HiSeq platform at NovoGene (UK). Successful ligation between the HPUF-gene carrying PCR-fragment and the plasmid vector generates ligation joints that in NGS will be sequenced from both strands, resulting in four kinds of sequence reads over the ligation joints (Figure 1b).

The raw sequencing read data were screened for the presence of the four expected sequences for each gene. To identify these reads, we generated in silico a list of ligation-joint covering sequences that included 15–25 nucleotides of specific sequence from both sides of the ligation joint (Figure 1b). The number of reads over the joints (joint-reads) reflects the number of intact and correctly ligated genes in the samples. The relative number of joint-reads of a specific gene was then calculated as a percentage of the total joint-reads in the sample. A toxic gene can be identified by a significant reduction in the relative number of the gene-specific joint-reads in the transformant plasmid pool compared to the relative number in the pooled ligation mixture. The complete pipeline of the bioinformatics analysis is described in detail in Table S5. The sequence analysis was performed using the Puhti computer at CSC (the Finnish Centre for Scientific Computing).

### 2.9. Protein Function and Sequence Analysis

The predicted functions and structures of protein sequences were obtained by modeling the protein sequence with Phyre 2 software [30] and aligning against protein sequence databases with BLASTx [31,32].

### 2.10. Confirmation of Toxicity by Growth Curve Analysis

The putative toxHPUF-encoding genes were selected to further confirm the toxicity. Genes were cloned into the multiple cloning site of the arabinose-inducible plasmid pBAD30. Plasmid and genes were digested with the *Kpn*I and *Xba*I enzymes or *Sph*I (Thermo Fisher Scientific, Waltham, MA, USA) if an internal *Xbal* site was present in the insert. An aliquot of each ligation mixture was transformed into electrocompetent *E. coli* DH5α cells as described earlier. Transformant colonies were selected and further grown overnight in an LB medium supplemented with ampicillin and glucose (0.2% *w/v*).

The constructs were confirmed by Sanger sequencing at the Finnish Institute for Molecular Medicine (FIMM). After overnight incubation, the cells were washed with M9t minimal medium and 10 µL was inoculated into the M9t medium supplemented with ampicillin and either glucose (0.2% *w/v*) or arabinose (0.2% *w/v*). Bacterial cells were distributed to Bioscreen Honeycomb plates and the OD_600_ was measured every hour for 20 h with Bioscreen C MBR (Oy Growth Curves Ab Ltd., Helsinki, Finland). Average ODs and standard deviation were measured from triplicate wells of three biological replicates per gene. As controls, *E. coli* strains carrying plasmids containing phage φR1-RT toxic or non-toxic (*g137* and *g150*) genes, or with the empty vector were used.

## 3. Results

### 3.1. fHy-Eco03 Host Range and Growth Characteristics

Phage fHy-Eco03 was isolated from a municipal sewage sample collected in Hyvinkää, Finland, using human *E. coli* isolate #5509 (Table 1) as the host. The genome size (57 kb), size of the prolate capsid (86 nm long), and the length of the tail (103 nm) of fHy-Eco03 (Figure S2) are characteristics of a dwarf Myovirus.

The host range experiments revealed that fHy-Eco03 has a relatively narrow host range, as it was able to produce clear lysis only in two (#5509 and #5517) of the 50 tested clinical strains (Table S1). In addition, strain #5520 was infected with low efficiency of plating (EOP); in eight strains (e.g., #5522), turbid plaques were produced. It should be noted that the clear plaques that fHy-Eco03 formed in its original host strain were small (Figure S3). Of note, fHy-Eco03 was found to infect a recent *E. coli* isolate from a patient suffering from a chronic urinary tract infection, and it was used in compassionate phage therapy treatment.

The killing efficiency of fHy-Eco03 was examined with four *E. coli* strains. The strains were infected in liquid culture at different MOI values, and the bacterial growth curves were determined. The results showed that fHy-Eco03 infected efficiently strains #5509, #5517, and, to our surprise, also strain #5522, and prevented growth in the cultures for 6–7 h after which the growth of apparently phage-resistant mutants became evident (Figure S4). No growth inhibition was seen with the low EOP strain #5520.

### 3.2. General Genomic Features of fHy-Eco03

The genome sequence was assembled using 250,000 reads with an average whole-genome coverage of 646x. The length of the double-stranded DNA genome is 57,011 bp with a GC-content of 45.1%. Restriction digestions were carried out with 10 different enzymes that had between 3 and 14 recognition sites in genomic sequence (Figure S5). Only *Sca*I was able to digest the DNA and the generated restriction fragments accommodate perfectly the in silico-predicted fragments (Figure S5). The methylation sensitivity or dependence of the used restriction enzymes could not explain the failure of the other enzymes to digest the DNA (Figure S5), suggesting that DNA carries an unknown modification that blocks the enzymes. Future work is required to elucidate the nature of the modification. Direct terminal repeats of 3699 bp were identified from the genome ends by the PhageTerm tool [21] (Figure S6). The terminal repeats were predicted to contain 11 genes.

**Figure 2:**
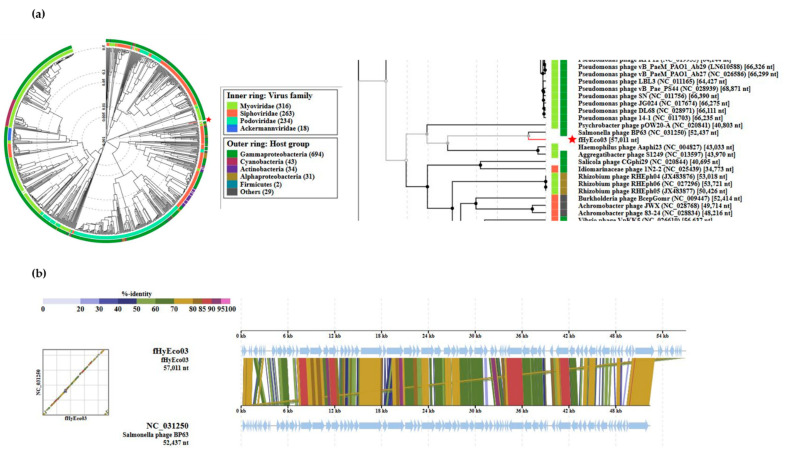
Position of fHy-Eco03 in the Phage Proteomic Tree generated by VIPTree [33]. (**a**) At left, a circular proteomic tree of prokaryotic dsDNA viruses colored by indicated virus families and host taxonomic groups. At right, part of the rectangular presentation of the proteomic tree showing the closest related phages to fHy-Eco03. The location of fHy-Eco03 in both is indicated by the red asterix. (**b**) The genomic alignment of fHy-Eco03 and the *Salmonella* phage BP63. Note the absence of the 3.6 kb terminal repeat in the BP63 sequence.

The fHy-Eco03 genome is predicted to encode for 81 genes with ATG as an initiation codon for 74 of the predicted genes, followed by TTG for four and GTG for three predicted genes. The overall organization of the genome (Figure S7) is almost identical to closely related phages (Figure 2 and Figure S8). Apart from the terminal repeats that encode 11 predicted genes, the fHy-Eco03 genome is bipartite, with genes from *g12* to *g48* transcribed in the forward direction and genes from *g49* to *g78* from the reverse strand. Close to the right terminal repeat, *g79* and *g80* are again transcribed in the forward direction in close proximity to *g01* and *g02* of the right terminal repeat. Of note, the proteomic analysis identified with high confidence peptides encoded in an open reading frame inside gene *g53*; we named this extra gene, *g53.1* (Table S6, Figure S7).

Initially, based on BLASTp search, most of the predicted gene products of fHy-Eco03 were annotated as HPUFs with no similarity to characterized proteins. Purified fHy-Eco03 particles were analyzed using the LC-MS/MS to identify the phage-particle-associated proteins (PPAPs), and altogether, 48 phage proteins were identified as PPAPs (Table S6). As the genome encodes 81 predicted gene products, and 48 were assigned as PPAPs, of the remaining 33 gene products, 25 were identified as true HPUFs since Gp23, Gp26, Gp40, Gp50, Gp54, Gp64, Gp65, and Gp72 showed homology to known proteins in HHpred analysis (Table S2).

The VIPtree server [33] was used to generate a phylogenetic tree indicating that the phage is most closely related to *Salmonella* phage BP63 (Figure 2). Whole genome nucleotide sequence megablast searches were carried out to identify the closely related phages from databases. Their phylogenetic relationships were determined using the Victor tool [28] demonstrating that fHy-Eco03 positions close to a number of other *Salmonella* phages (Figure S8). This allowed us to classify fHy-Eco03 as a member of the Rosemountviruses [34]. Genome alignment of fHy-Eco03 with the *Salmonella* phage BP63 is shown in Figure 2b, demonstrating the close relatedness over the full lengths of the phage genomes. The calculated coding density for fHy-Eco03 was 83.4%, which is a bit lower than that seen in other members of the Rosemountviruses or the also closely related Loughboroughviruses [34]. As no genes encoding integrase, lysogeny, virulence or antimicrobial resistance-associated proteins were identified in the genome of phage fHy-Eco03, based on current opinion, the phage is considered safe for use in phage therapy.

### 3.3. Identification of toxHPUFs by Plating Efficiency and NGS-Based Screening Assays

**Figure 3:**
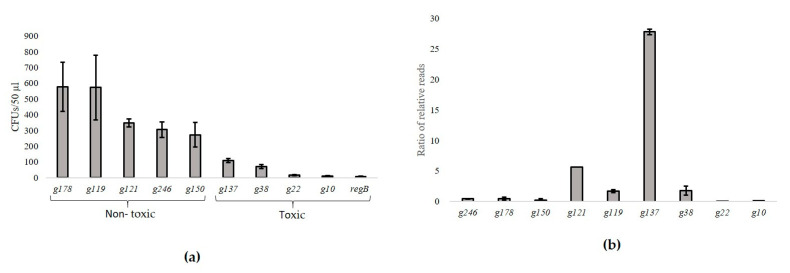
Comparison of toxicity screenings with control genes. (**a**) The plating efficiency assay shows the average numbers (±SD) of transformants for non-toxic (*g178*, *g119*, *g121*, *g246* and *g150*) and toxic (*g137*, *g38*, *g22*, *g10* and *regB*) control genes (Table S3), based on two biological replicates with triplicate platings for each gene (Table S7). (**b**) In the NGS screening assay, the percentages of the joint-reads for each gene were calculated relative to the total joint-reads for both the pooled ligation mixture and the pooled plasmids from the transformants. Shown are the ratios of the percentages in the plasmid DNA of transformants versus those in the ligation mixture. A toxic gene should produce a ratio significantly below 1 (see Table S8 for details). The bars show for means ± SD for two replicate transformations.

Some of the HPUFs of fHy-Eco03 potentially encode toxic proteins. Previously, the plating efficiency assay was used to identify toxic HPUFs [8,10], but this assay is not optimal in terms of reproducibility and efficiency. Therefore, we set up an NGS-based screening assay to identify toxic proteins. Known toxic and non-toxic phage gene products, five each originating from *Escherichia* phage T4, *Yersinia* phage φR1-RT, and *Klebsiella* phage fHe-Kpn01 (Table S4) were first tested in the plating efficiency assay to assess whether the previously published results were reproducible [8,10]. This was indeed the case as the non-toxic controls produced between ~200 (*g150*) and ~700 (*g178*) transformants, and the toxic controls between 5 (*regB*) and ~100 (*g137*) transformants (Figure 3, Table S7).

The relative toxicities of the same genes were then tested with the NGS screening assay as illustrated in Figure 1a. The control genes were ligated independently into the cloning vector. The ligation mixtures were then pooled together, and an aliquot was withdrawn for NGS, and then the ligation pool was electroporated into *E. coli*. The obtained transformants were pooled and the isolated plasmid DNA was subjected to NGS. The ligation joint-reads were then extracted from both types of DNA samples as described (Table S5). The proportions of gene-specific joint-reads for each gene among all extracted reads were calculated for both types of samples. Theoretically, a toxic gene would not establish itself as a plasmid among the transformants, and, therefore, would have no or very few joint-reads among the extracted plasmid DNA join-reads. Conversely, the relative proportion of the joint-reads of non-toxic genes could even increase. For each gene, the joint-read percentage in the plasmid sample was divided by the joint-read percentage in the pooled ligation mixture (Figure 3b, Table S8). Unfortunately, no *regB* specific joint-reads were detected in any of the samples, suggesting that its ligation mix was either destroyed or accidentally omitted from the pool. The joint-read ratios of the toxic control genes *g10*, *g22*, *g38*, and *g137* were 0.15, 0.02, 1.8, and 27.2, respectively, and those of the non-toxic control genes *g119*, *g121*, *g150*, *g178*, and *g246* were 1.68, 5.63, 0.19, 0.48, and 0.44, respectively (Figure 3b, Table S8). Based on these results, the NGS screening would have identified only the *g10* and *g22* gene products as toxic candidates.

When preparing the ligation mixtures, each ligation reaction contained the same amount of vector DNA, and equimolar amounts of PCR-fragments. Therefore, in an optimal situation, the numbers of gene-specific joint-reads should be equal for each gene in the pooled ligation mix—in this experiment with ten genes, 10%. However, the joint-read percentages in the original ligation mixture varied between 0.48% for *g137* and 23.11% for *g246* (Table 2), indicating that ligation efficiencies between the genes varied a lot. As the vector DNA in all ligations were the same, a possible explanation for the variation has to be in the properties and/or the quality of the PCR-fragments that influence the ligation efficiency.

Even keeping this in mind, the unexpectedly high ratio of *g137* cannot be accounted for, especially as it demonstrated moderate toxicity in the plating assay (Figure 3a) and apparently no toxicity in the NGS screening assay. Analysis of the aligned sequence reads from the plasmid samples against the gene *g137* sequence demonstrated that the cloned gene *g137* sequence was intact. Therefore, keeping in mind that the pooled ligation mixture included altogether six phage φR1-RT genes, there is a possibility of a co-transformation of the host bacteria with two or more different plasmids at the same time. In such a case, in ordinary situations without any selective pressure, only one plasmid per offspring of the co-transformant would be carried further. In the case of transformants carrying the *g137-*containing plasmid, the toxicity of Gp137 would generate a selective pressure. Thus, a plausible explanation for the observed phenomenon could be that the *g137* carrying transformants was co-transformed with a plasmid carrying a gene encoding an anti-toxin. Thus, when inspecting the joint-read number percentages, the co-transformed gene should have at least the same percentage as *g137* (13.16%) and perhaps even higher, as it would also survive as an individual non-toxic transformant. Only two of the φR1-RT genes fulfilled this requirement, *g119* and *g121* that have joint-read number percentages of 19.53 and 15.74, respectively (Table 2). Interestingly, the increase in the percentage between the ligation and plasmid samples is close to 13% for both *g121* and *g137,* while it is only 8% for *g119*. Whether Gp137 and Gp119 or Gp121 make a toxin and anti-toxin system can be easily tested by co-transforming those genes together to a host bacterium.

### 3.4. Screening the fHy-Eco03-Encoded HPUF Genes

The plating efficiency assay was used in parallel with the NGS screening assay to screen 23 HPUF-encoding genes of fHy-Eco03. In addition, 9 more HPUF-encoding genes were screened using the NGS assay only (Table 3, Table S9 and S10). For the plating efficiency assay, the number of transformants was normalized to the non-toxic gene *g178,* which was included as a control in each batch of electroporations (Table 3). The ratios ranged from 0.16 of *g05* to 1.71 of *g48.* In addition to *g05,* the ratios of six genes (*g52*, *g64*, *g51*, *g68*, *g10*, and *g65*) were below 0.6, indicating a potentially toxic gene.

For the first batch of the NGS screening assay, the ligation mixtures of the 23 HPUF-encoding genes of fHy-Eco03 were pooled together and electroporated to the host bacteria. In the second batch, the ligation mixtures of the remaining nine HPUF-encoding genes were pooled together and electroporated. Details of both batches are given in Table S10, and summarized in Table 3. Theoretically, each gene should be represented in the first ligation batch with a 4.3% share, and in the second batch with an 11.1% share. The results show that the shares ranged between 0.89 and 11.44% for batch 1, and 0.96 and 36.90% for batch 2, indicating that also here, the genes had not ligated with equal efficiency, again possibly due to variation in the properties and quality of the PCR products.

The ratios between the ligation mix and the plasmid DNA joint-read percentages ranged between 0.003 and 7.32 (with a mean ± SD of 1.76 ± 1.78). As the ratio below 1.0 should indicate toxicity and taking into account the variation in ligation efficiencies, we chose to consider genes with ratios around or below 0.5 as toxic candidates. Altogether, seven genes were in this group, with four of them also tested in the plating efficiency assay (Table 3). Considering both these screening assay results, the gene products of *g05*, *g10*, *g23*, *g40*, *g51*, *g52*, *g58*, *g64*, *g68*, *g73*, and *g77* demonstrated some toxicity in one or both of the assays, with only Gp05 clearly presenting the most toxicity in both assays.

The numbers of NGS joint-reads for each alignment are displayed in detail in Table S10. While sequence coverages of complementary fragments were equal in the DNA samples of the plasmids isolated from pooled transformants, the corresponding reads from the ligation mixture varied substantially. The difference between read coverages of complementary fragments varied consistently up to a thousand-fold, as in some cases, only a few to zero reads were obtained (Tables S8 and S10). Apparently, due to the short size of the gene fragments, there is a bias in the generation of the DNA library for NGS that distorts the distribution of the sequence reads, favoring those that start from the plasmid side.

### 3.5. Gp05 Showed Toxicity among the Tested toxHPUF Candidates of fHy-Eco03

**Figure 4:**
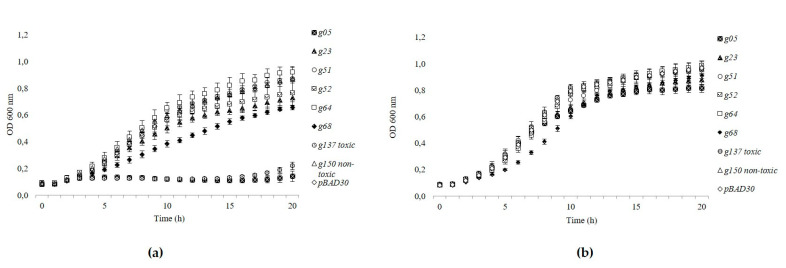
Growth curves of *E. coli* DH5α cells containing pBAD30 plasmid with cloned fHy-Eco03 candidate toxHPUF encoding genes, during (**a**) arabinose induced or (**b**) glucose inhibited expression. Average OD_600_ values were determined from triplicate wells of three biological replicates.

The growth-inhibition potential of the phage fHy-Eco03 gene products Gp05, Gp23, Gp51, Gp52, Gp64, and Gp68 was tested with a growth curve analysis by cloning each gene into an arabinose-inducible pBAD30 vector. Absorbances (OD_600_) measured during arabinose induced or glucose inhibited expression during a 20-h incubation are presented in Figure 4. The growth of the arabinose-induced Gp05 expressing cells was inhibited in a similar manner to that of the toxic control Gp137 of φR1-RT [8], thus confirming the toxicity of Gp05 to *E. coli*. The other gene products of fHy-Eco03 demonstrated, at best, only moderate growth inhibition, exemplified by Gp68 (Figure 4a). The glucose-repressed cultures all showed a steady exponential growth (Figure 4b). It must be noted that the toxicity screen of fHy-Eco03 HPUFs was not exhaustive, as all the possible toxHPUF candidates with ratios below 0.5 identified in Table 3 were not included in this experiments.

### 3.6. Functional and Structural Analysis of Gp05

**Figure 5:**
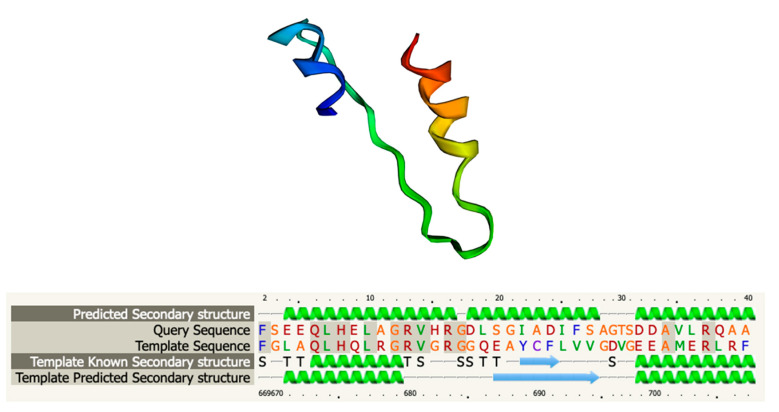
Protein structure and sequence alignment of Gp05, modeled with Phyre2 software.

Gp05 is predicted to be an 81 amino-acid-long protein that is 68–78% identical to five HPUFs of closely related *Salmonella* phages, with the highest identity to Gp60 of phage ZCSE2 (Accession number MK673511, Figure S8). In addition, the N-terminal half of Gp05 showed some similarity to the C-terminal parts of the ca. 700 amino-acid-long ATP-dependent helicases of several different bacteria. Interestingly, modeling with Phyre2 software [30] resulted in a model with 73.60% confidence for the 39 N-terminal residues of Gp05 against the residues 670–710 of the 780 amino-acid-long helicase RecG (PDB 1GM5) (Figure 5) of *Thermotoga maritima* [35]. These residues are located in the C-terminal domain 3 of the RegG protein that is involved in ATP binding, and are very close to the region that interacts with the DNA structure [35].

## 4. Discussion

In this study, we describe a new NGS-based screening assay for the detection of bacteriophage-encoded toxic proteins. The performance of the NGS screening assay was compared with the previously used plating assay by screening known toxic and non-toxic genes and the HPUFs of phage fHy-Eco03. Among the fHy-Eco03 HPUFs, the Gp05 was found to be toxic in both the screening assays and the growth inhibition assay. The amino acid sequence of Gp05 showed 68–78% identity to five HPUFs of *Salmonella* phages. Despite fHy-Eco03 being isolated in *E. coli*, *Salmonella* and *Escherichia* bacteria are closely related species; indeed, it is not surprising that the structure and function of Gp05 and the obtained proteins were similar. The N-terminal half of Gp05 was best modeled by Phyre2 against the fold present in RecG, an ATP-dependent helicase. The RecG protein was first described for *E. coli.* It functions in resolving stalled replication forks, and RecG homologs can be found in a majority of bacterial species. RecG is an essential, multifunctional protein involved in several DNA repair and replication-related pathways, thus it would be a good target for the bacteriophage-mediated arrest of cellular functions [36,37,38]. It is tempting to speculate that the Gp05 toxicity could be mediated by its ability to interfere the RecG activity. To further characterize the structure, mechanisms, and cellular targets of Gp05, comprehensive protein–protein interaction studies are required. This involves expression and purification of the toxic protein, which is often challenging because of possible toxic and deleterious effects to the bacterial production host. In such a case, as an alternative, yeast and plant cells could be used as the production host, or the toxic properties and targets could be identified through an in vitro translation approach.

Hypothetical proteins have previously been screened with interaction studies between known bacterial targets and hypothetical proteins [11], or by cloning shotgun sheared genomes [9] and annotated hypotheticals [6] into inducible expression vectors. Van Den Bossche et al. discovered eight bacteriotoxic proteins by screening 32 hypothetical proteins of seven different phages against cellular targets of *Pseudomonas aeruginosa* by utilizing affinity chromatography [11]. However, since toxic proteins are discovered through interactions with specific targets, proteins with unknown cellular targets are inevitably overlooked. In a study conducted by Singh et al., seven Siphovirus genomes were sheared and cloned to inducible vectors and screened for toxic effects in two *Mycobacterium* strains [9]. Two toxic proteins and two synthetic peptides were discovered with this approach. The peptides, however, had unnatural ORFs, pointing out the disadvantage of this method. By using sheared genomes, the risk of producing vector constructs with either fragmented ORFs or incorrectly oriented genes increases and these can easily be interpreted as false positives or negatives. A similar expression vector-based approach was used by Liu et al. with the difference of first identifying true hypotheticals for screening. With this method, 31 protein families with toxic properties were identified from 27 different *Staphylococcus* phages [6].

The plating assay used in the present study as a reference method is similar to the approach used by Liu et al. [6]. Since many PPAPs are not identified by bioinformatics tools, we first identified them by LC-MS/MS analysis carried out on purified phage particles, and only then, the remaining HPUFs were screened for toxic ones using the plating efficiency assay [8,10]. The HPUF-encoding genes are cloned to an expression vector and transformed into electrocompetent *E. coli* cells by electroporation. Toxic properties were identified by comparing transformation efficiencies against cells transformed with a vector containing a non-toxic control gene. Although this method minimizes the amount of genes to be screened, it still has several drawbacks that reduce the reliability and repeatability of the results, as many steps of the screening approach are hard if not impossible to control and standardize. Firstly, surviving transformants can be generated by undigested or self-ligated plasmids, or by gene fragments that are incorrectly ligated to the vector plasmid. Secondly, electroporation conditions in which the cells are transformed cannot be reliably controlled, thus are not ideal for quantitative experiments such as this. Finally, the results are affected by variations in temperature, pipetting, and plating. In order to increase the reliability and reproducibility, the HPUFs had to be screened with several replicate batches together with control genes. While this did decrease the variation, the required amount of resources and time increased almost exponentially.

The performance of the NGS screening assay was first tested by conducting an experiment with five non-toxic and five toxic genes of phages fHe-Kpn01 [10], T4 [39], and φR1-RT [8]. The plating efficiency assay confirmed that RegB of T4, Gp137 of φR1-RT, and Gp10, Gp22, and Gp38 of fHe-Kpn01 were toxic. In the parallel NGS-based screening assay, however, only Gp10 and Gp22 showed toxicity. The *regB* ligation, due to an unknown technical error, dropped out from the pooled ligation mixture, thus leaving the unexpected results of Gp38 and Gp137 unaccounted for. The possibility of co-transformation of an anti-toxin expressing gene was already discussed for Gp137 (Section 3.4), and this could also be the case for Gp38. As a conclusion, it has to be noted that the interpretation of the NGS-based assay results may face unknown challenges. In the following NGS-based assays, we screened the HPUF-encoding genes of phage fHy-Eco03. The results of the plating efficiency and the NGS-based screening assays agreed to great extent (Table 3), and the same gene products presented toxic and non-toxic effects in both assays, with *g05* identified as most toxic in both assays (Table 3), as well as later in the growth inhibition assay (Figure 4). Despite grossly similar results, the variation between replicates in the NGS screening assay was substantially lower compared to that of the plating assay (Table S9).

The NGS-based screening assay was designed to increase reliability and significantly reduce the time and resources required for the hands-on lab work and further analysis of the results. This was enabled by performing the lab work and bioinformatics analysis of the preliminary screening simultaneously for all genes. As seen from the results, variation between the obtained sequence coverages of replicate transformations and even biological replicates was very small.

In conclusion, we have introduced here the NGS-based efficient screening assay for the identification of toxHPUFs. The performance of the assay still relies on very careful laboratory work when preparing the plasmid vector and the PCR-amplified DNA-fragments for ligations.

## Figures and Tables

**Table 1:** Bacteriophages, bacteria, and plasmids used in this study.

Bacteriophages and Bacteria	Comments	Reference
***Bacteriophages***		
φR1-RT	PCR template for phage φR1-RT genes	[14]
T4	PCR template for phage T4 genes	[12]
fHe-Kpn01	PCR template for phage fHe-Kpn01 genes	[10]
fHy-Eco03	PCR template for phage fHy-Eco03 genes	This work
***E. coli strains***		
#5509	Human blood culture isolate	This work
DH10B	Cloning host	[15]
DH5α	Cloning host	[16]
DH10B/pU11L4	Source of plasmid pU11L4	[8]

**Table 2:** The gene-specific joint-read percentages from total joint-reads extracted from the NGS sequence reads of the pooled ligation mixture and the pooled transformant plasmid DNA, and their corresponding ratios.

Gene	Joint-Read Number %		Ratio
	Ligation Mixture	Transformant Sample	
*g10*	0.86	0.13	0.15
*g22*	3.10	0.07	0.02
*g38*	14.84	26.83	1.81
*g119*	11.70	19.53	1.67
*g121*	2.79	15.74	5.63
*g137*	0.48	13.16	27.2
*g150*	21.38	3.81	0.18
*g178*	21.74	10.58	0.49
*g246*	23.11	10.17	0.44
*Total*	100	100	

**Table 3:** Comparison of NGS and plating assay results for the fHy-Eco03 HPUF encoding genes. For the NGS screening assay, shown are the percentages of the gene-specific joint-reads in the ligation mix and the plasmid DNA samples, and the plasmid/ligation ratios calculated based on them. The data have been determined from two biological replicates of the pooled ligation mixtures and two replicate transformations per mixture (details in Tables S9 and S10). The average fractions in the plating assay were determined by dividing the average number of transformants for each gene with that of the non-toxic control gene *g178* processed in the same batch. The plating assay was carried out with two biological replicates and triplicate platings. Potentially toxic ratios are highlighted in grey. Genes chosen for further screening are given in bold.

NGS Screening Assay Results					Plating Assay Results		
Gene	% Reads in Ligation Mix	% Reads in Plasmid DNA	Ratio	SD	Gene	Fraction of *g178*	SD
*g01*	1.31	3.52	2.69	0.114	*g01*	0.680	0.166
*g03*	1.26	5.33	4.22	0.546	*g03*	1.003	0.015
*g04*	0.89	2.93	3.30	0.167	*g04*	0.672	0.035
*g05*	3.36	0.01	0.003	0.001	*g05*	0.164	0.003
*g06*	1.46	3.56	2.43	0.328	*g06*	0.684	0.193
*g08*	1.28	3.85	3.01	0.196	*g08*	0.658	0.130
*g09*	2.80	4.30	1.53	0.027	*g09*	0.827	0.145
*g10*	1.08	1.63	1.50	0.334	*g10*	0.463	0.186
*g11*	1.07	2.97	2.78	0.043	*g11*	0.901	0.250
*g13*	2.87	5.71	1.99	0.202	*g13*	1.179	0.428
*g15*	8.64	5.21	0.60	0.074	*g15*	1.104	0.349
*g17*	7.79	5.49	0.71	0.014	*g17*	0.868	0.224
*g18*	36.90	41.23	1.12	0.017	--	--	--
*g23*	12.09	3.28	0.27	0.005	*g23*	0.683	0.200
*g26*	19.23	12.80	0.67	0.055	--	--	--
*g40*	11.47	3.63	0.32	0.036	--	--	--
*g48*	3.40	15.39	4.52	0.288	*g48*	1.711	0.106
*g50*	2.67	4.08	1.53	0.099	*g50*	0.746	0.234
*g51*	0.91	5.41	5.96	0.072	*g51*	0.440	0.180
*g52*	6.75	3.81	0.56	0.033	*g52*	0.332	0.089
*g58*	0.96	0.33	0.34	0.056	--	--	--
*g59*	2.36	17.27	7.32	1.827	--	--	--
*g61*	2.71	9.41	3.47	0.047	--	--	--
*g64*	7.91	2.07	0.26	0.024	*g64*	0.332	0.089
*g65*	5.39	5.40	1.00	0.049	*g65*	0.515	0.140
*g68*	1.31	1.06	0.81	0.034	*g68*	0.451	0.077
*g69*	8.18	5.62	0.69	0.023	*g69*	0.795	0.237
*g71*	11.59	10.57	0.91	0.059	--	--	--
*g72*	4.08	2.43	0.59	0.064	--	--	--
*g73*	10.71	2.34	0.22	0.019	--	--	--
*g76*	3.94	4.60	1.17	0.068	*g76*	1.296	0.505
*g77*	11.44	4.77	0.42	0.051	*g77*	0.974	0.481

## Data Availability

Data is contained within the article and Supplementary Material.

## Supplementary Materials

The following are available online at https://www.mdpi.com/article/10.3390/v13050750/s1, Figure S1: Map of plasmid pU11L4; Figure S2: Transmission electron micrograph of a negatively stained fHy-Eco03 particle; Figure S3: Plaque morphology of *E. coli* strain #5509 infected with fHy-Eco03; Figure S4: Growth curves of *E. coli* strains #5509, #5517, #5520, and #5522 infected with fHy-Eco03; Figure S5: Restriction enzyme digestion analysis of fHy-Eco03 DNA; Figure S6: Detection of genome ends of fHy-Eco03; Figure S7: Genomic map of fHy-Eco03; Figure S8: Genomic comparison of fHy-Eco03; Table S1: *E. coli* strains used to determine the host range of fHy-Eco03; Table S2: Annotation of the predicted gene products of fHy-Eco03; Table S3: LC-MS/MS analysis of the proteome of the fHy-Eco03 virion particles; Table S4: Primers to amplify control genes encoding toxic and non-toxic gene products; Table S5: Primers used to amplify the HPUF genes of fHy-Eco03; Table S6: Workflow for the NGS assay bioinformatics; Table S7: Results from plating assay of control genes; Table S8: Results of the NGS assay with control genes; Table S9: Plating assay results of 23 HPUF-encoding genes of fHy-Eco03; Table S10: NGS screening of phage fHy-Eco03 HPUF encoding genes.
