# Supplementary material for: Screening of Bacteriophage Encoded Toxic Proteins with a Next Generation Sequencing-Based Assay
Source: Viruses. 2021 Apr 24;13(5):750. doi: 10.3390/v13050750 (PMC8145870; doi:10.3390/v13050750)
Supplement: Supplementary file 1 [file viruses-13-00750-s001.zip › viruses-1186473-supplementary.pdf]

viruses

MDPI

Supplementary Materials

Screening of bacteriophage encoded toxic proteins with a next generation sequencing based assay

Jutta Kasurinen[1], Cindy M. Spruit[1,†], Anu Wicklund[1,2], Maria I. Pajunen[1#], and Mikael Skurnik[1,2,#*]

1 Department of Bacteriology and Immunology, Medicum, Human Microbiome Research Program, Faculty of Medicine, University of Helsinki, 00290 Helsinki, Finland; jutta.kasurinen@helsinki.fi (J.K.), c.m.spruit@uu.nl (C.M.S.); anumaria.wicklund@gmail.com (A.W.); maria.pajunen@helsinki.fi (M.I.P.); mikael.skurnik@helsinki.fi (M.S)
2 Division of Clinical Microbiology, HUSLAB, University of Helsinki and Helsinki University Hospital, 00290 Helsinki, Finland
† Present address: Department of Chemical Biology & Drug Discovery, Utrecht Institute for Pharmaceutical Sciences, Utrecht University, 3584 CG Utrecht, the Netherlands
# Shared senior authorship
* Correspondence: mikael.skurnik@helsinki.fi; Tel.: +358 50 3360981

Supplementary Figures

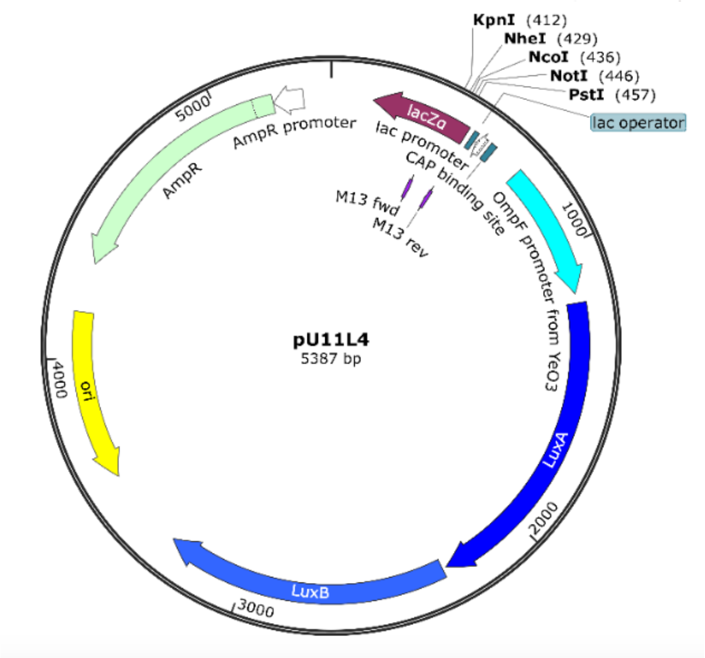

Figure S1. Map of plasmid pU11L4 [1].

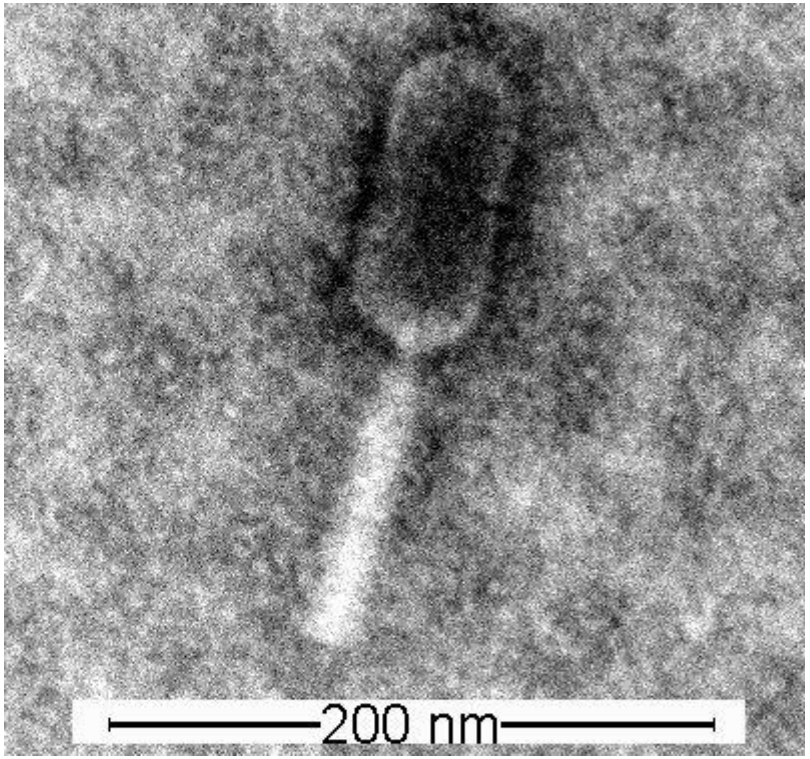

Figure S2. Transmission electron micrograph of a negatively stained fHy-Eco03 particle (capsid length 86 nm, tail length 103 nm, phage length 208 nm).

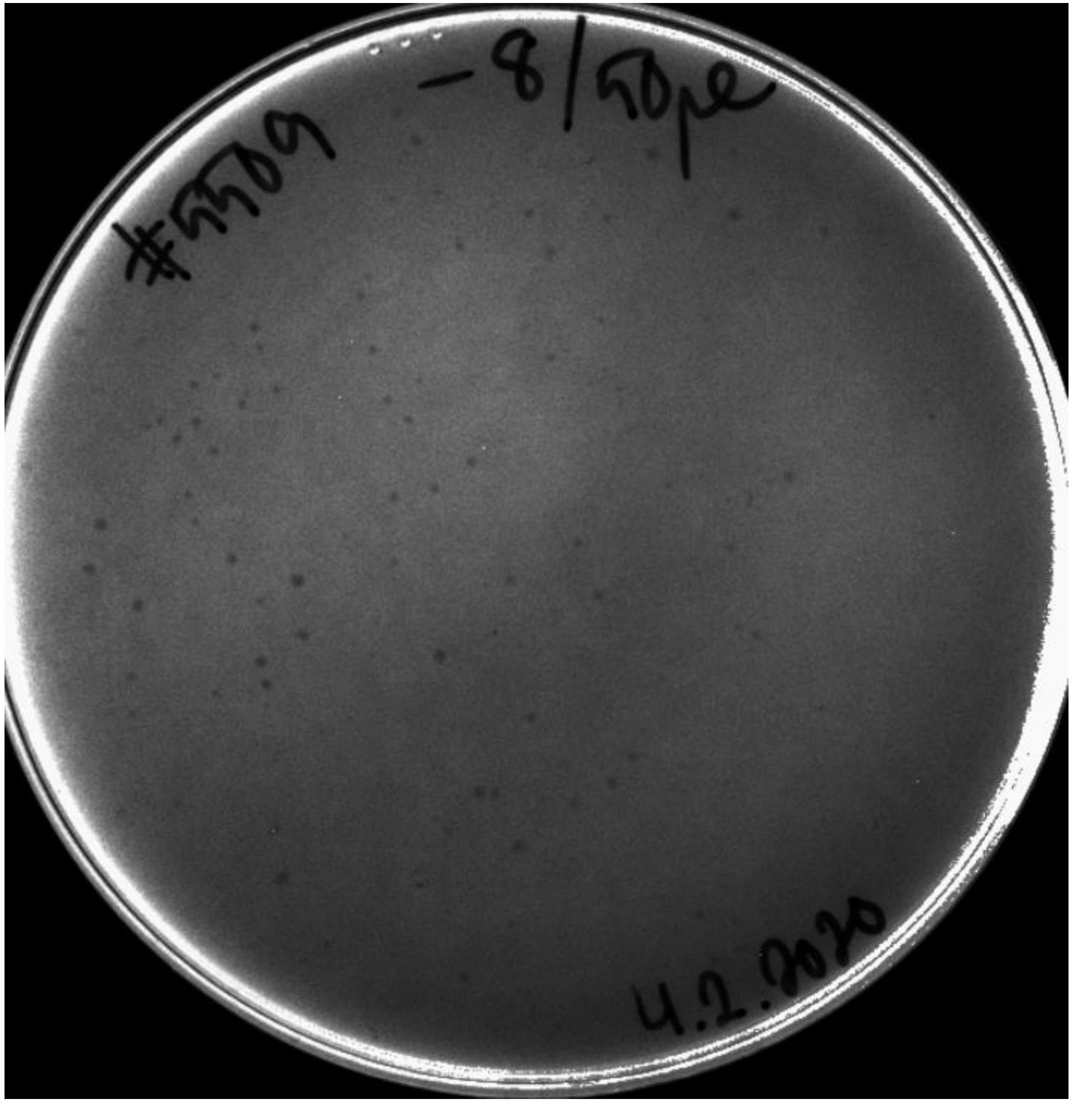

Figure S3. Plaque morphology of *Escherichia coli* strain #5509 infected with fHy-Eco03.

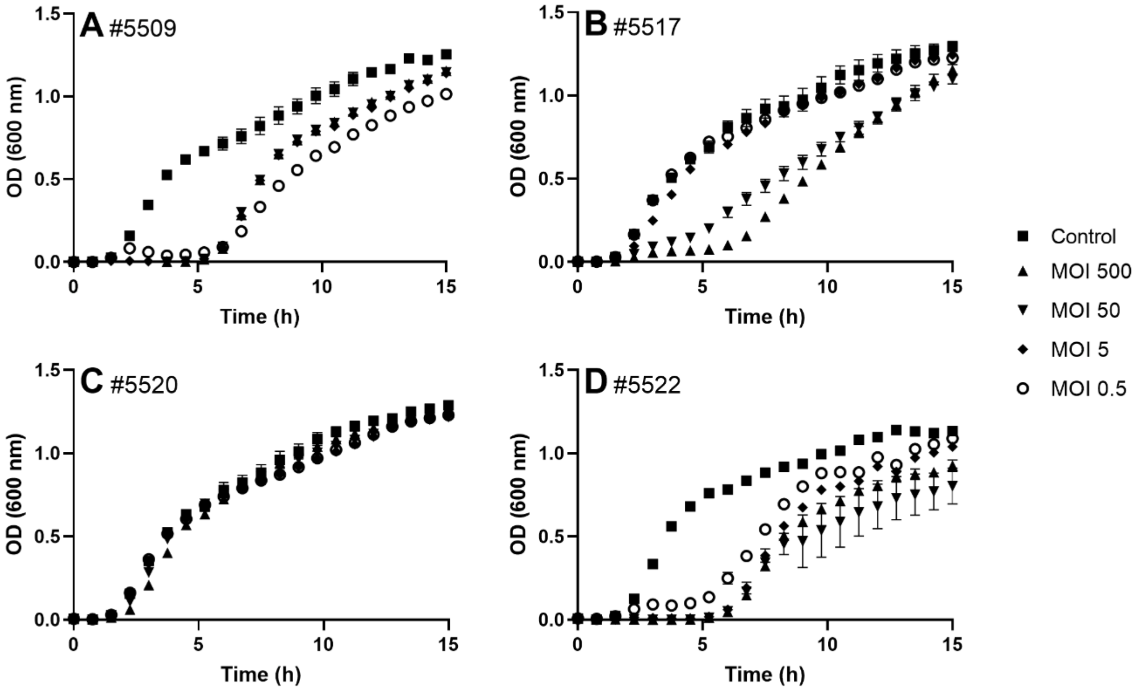

Figure S4. Growth curves of *Escherichia coli* strains #5509 (A), #5517 (B), #5520 (C), and #5522 (D) infected with fHy-Eco03. Bacteria were cultured in liquid LB medium at 37°C with different initial multiplicities of infections (MOIs). Each data point represents the average OD_{600} for three to four replicates and error bars represent the standard deviation (SD).

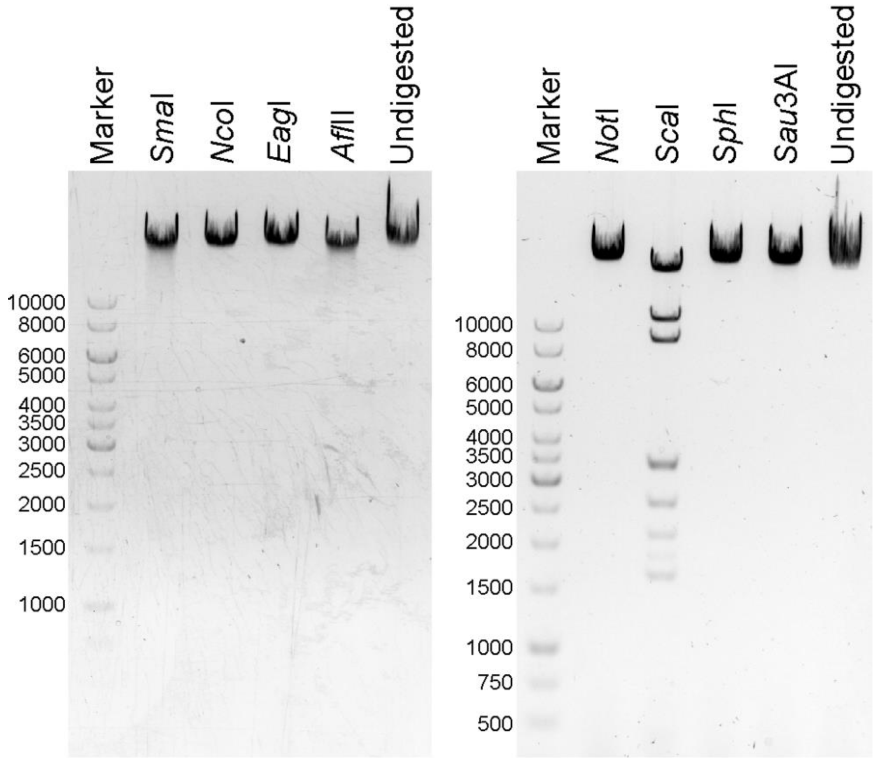

Figure S5. Restriction enzyme digestion analysis of fHy-Eco03 DNA. The DNA was digested with restriction endonucleases *Afl*II, *Eag*I, *Nco*I, *Not*I, *Sau*3AI, *Sca*I, *Sma*I, and *Sph*I and the digeted samples were loaded to the gel as indicated, along with undigested phage DNA and 1 kb DNA ladder (Marker). The *in silico* ScaI-digested genomic sequence gives the following fragments (bp): 24,830; 10,405; 8,539; 3,337; 3,260 (left end); 2,531; 2,074; 1,596; 439 (right end). For the used restriction enzymes, the numbers of predicted recognition sites in the genome are as follows:

**Enzyme**	**Recognition sequence**	***n* sites**	**Methylation sensitivity / dependence** (https://enzymefinder.neb.com/)
*Afl*II	CTTAAG	10	None/None
*Eag*I	CGGCCG	5	CpG blocked/None
*Nco*I	CCATGG	7	None/None
*Not*I	GCGGCCGC	1	CpG variable/None
*Sau*3AI	GATC	9	Some block/None
*Sca*I	AGTACT	8	EcoBI: blocked/None
*Sma*I	CCCGGG	3	CpG blocked/None
*Sph*I	GCATGC	14	EcoBI: blocked/None

fHyEco PhageTerm Analysis

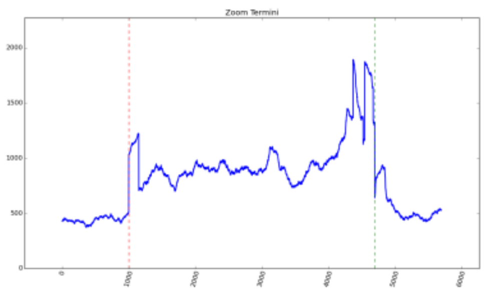

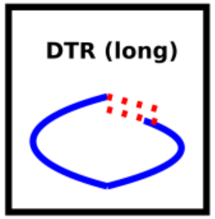

PhageTerm Method

Ends	Left (red)	Right (green)	Permuted	Orientation	Class	Type
Redundant	47595	51293	No	NA	DTR (long)	T5

Direct Terminal Repeats: 3699 bp

Strand	Location	T	pvalue
+	47595	0.66	1.47e-114
	51118	0.40	2.11e-26
	41280	0.10	1.30e-04
	23058	0.06	1.00e+00
	33934	0.06	2.11e-01
-	51293	0.68	1.03e-195
	51117	0.59	3.89e-149
	51453	0.07	1.67e-07
	35956	0.06	9.95e-01
	23478	0.06	1.00e+00

T (Start. Pos. Cov. / Whole Cov.)

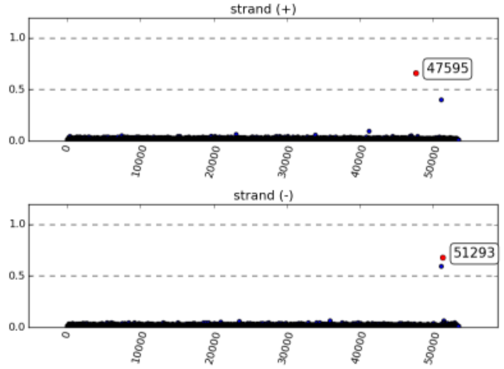

Figure S6. Detection of genome ends of fHy-Eco03. PhageTerm output (according to the PhageTerm method) showing major peaks in the ends of the genome on both positive and negative strands.

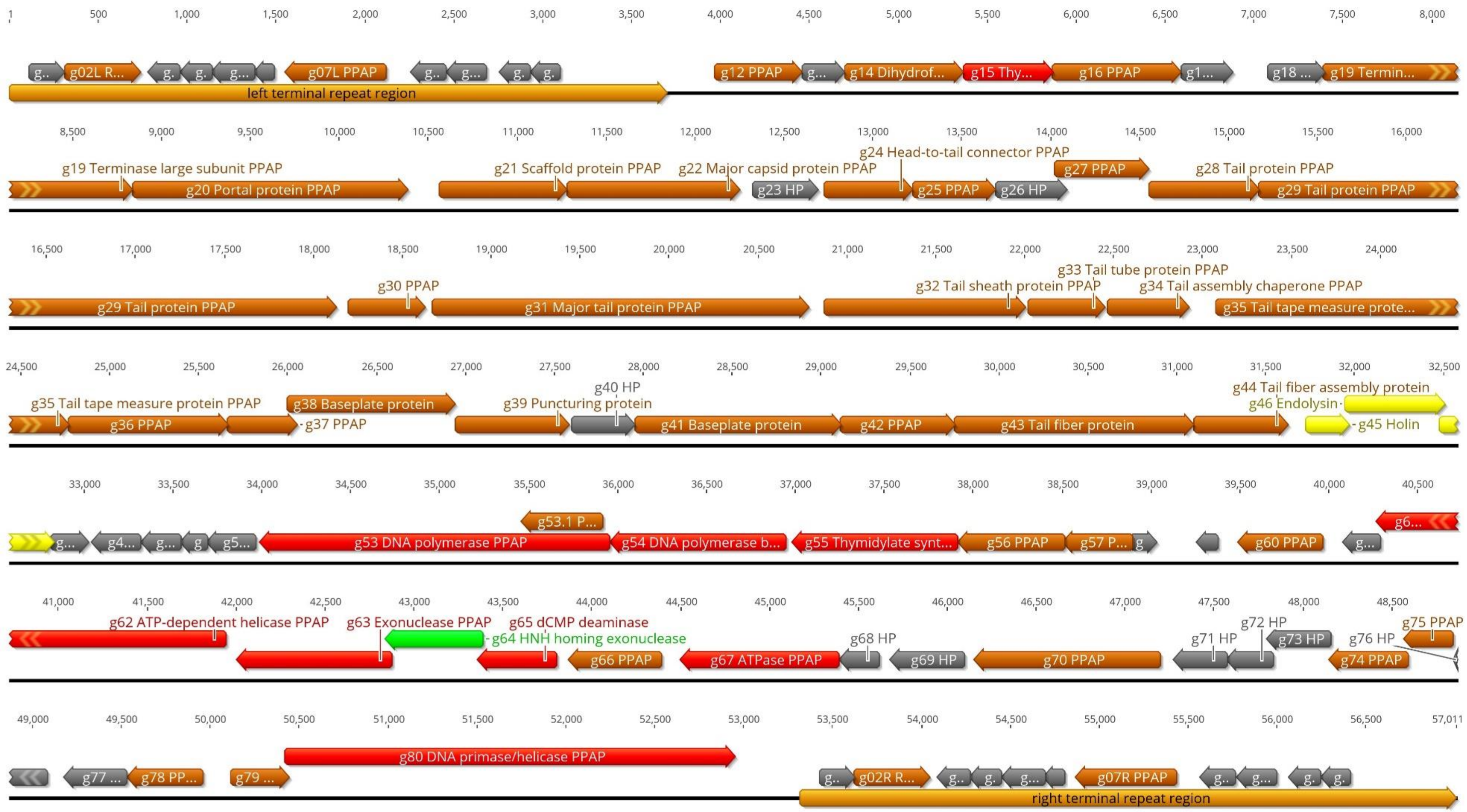

Figure S7. Genomic map of fHy-Eco03. Geneious Prime (version 2021.0.3) program was used for visualization. The genes encoding structural or phage particle associated proteins (PPAPs) are drawn in brown, nucleic acid metabolism associated enzymes in red, cell lysis associated proteins in yellow, endonuclease in green, and hypothetical proteins in grey. PPAP, phage particle associated protein; HP, hypothetical protein.

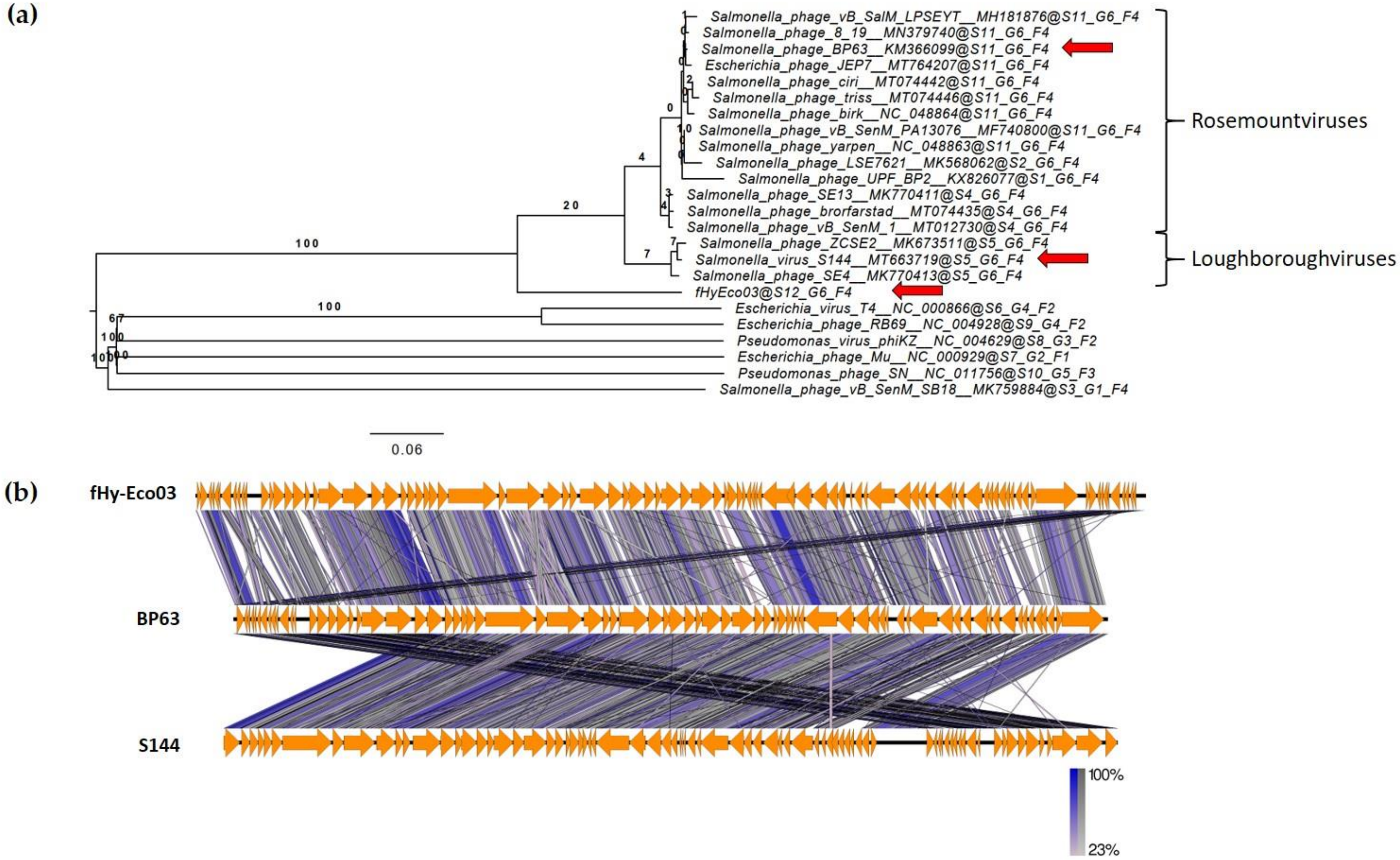

Figure S8. Genomic comparison of fHy-Eco03 **(a)** The phylogenetic genome-BLAST distance phylogeny (GBDP-D0) using VICTOR tool. Salmonella phage BP63 is the type phage for Rosemountviruses, and Salmonella phage ZCSE2 is the type phage for Loughboroughviruses, present in the ICTV virus taxonomy (release 2019). Some additional Myoviruses, especially those identified in the HHPRED analysis (Table S2) were included in the analysis. **(b)** An EasyFig generated comparison of the genomes of phages fHy-Eco03 (top), BP63 (middle) and S144 (bottom), indicated in panel (a) with red arrows, with different shades of blue indicating the identities between the genomes.

Supplementary Tables

Table S1. *Escherichia coli* strains used to determine the host range of fHy-Eco03. All the *E. coli* strains were isolated from human patient samples at the Clinical Microbiology Laboratory of the hospital district of Helsinki and Uusimaa, Finland. The Skurnik laboratory strain collection storage numbers are shown. Strains that produce extended-spectrum beta-lactamase (ESBL) are indicated. Strains that were infected by phage fHy-Eco03 are indicated as follows: ++, clear strong lysis; +/–, lysis but low EOP; -, no infection. The sensitivity of the strains indicated **in bold** to fHy-Eco03 was confirmed by determining infection growth curves using the Bioscreen C incubator (see Figure S4).

**Storage #**	**Strain**	**Origin**	**Antibiotic resistance**	**Sensitivity to fHy-Eco03**
5506	123794	Blood		–
5507	123789	Blood		–
**5509**	123781	Blood		++
5510	123780	Blood		–
5512	123770	Blood		–
**5517**	123745	Blood		++
5519	123741	Blood		–
**5520**	123740	Blood		+/- Turbid plaques
5521	123738	Blood		–
**5522**	123734	Blood		Turbid plaques
5625	13ME7768	Appendix abscess		–
	ME07799	Gall bladder fluid		–
5641	UB14789	Urine		–
5768	13MD3432	Rectal secretion	ESBL	–
	UB15006	Urine	ESBL	–
	KK08907	Amniotic fluid		–
	KK08908	Sacrum wound		–
	KP09927	Surgical wound		–
	UC14791	Urine	ESBL	–

5626	UF0545	Urine	ESBL	–
5759	EL3622	Feces	ESBL	Turbid plaques
5760	EL3615	Feces	ESBL	Turbid plaques
5761	W6215	Blood	ESBL	Turbid plaques
5762	EL3628	Feces	ESBL	–
5763	UC14886	Urine	ESBL	–
5764	UD14891	Urine	ESBL	–
	KP09910	Abscess		–
5627	UC 14827	Urine	ESBL	–
5628	UE14765	Secretion	ESBL	–
5765	UF05451	Urine	ESBL	–
5629	ME8280	Abdominal punction		–
5766	13KP10517	Abscess in gluteal cleft		–
5767	13KP10501	Foot surgical wound		–
5630	TS07654	Thigh wound		–
5631	KK4340	Foot sole wound		–
5632	UB15026	Rectum mucus	ESBL	–
	UB15626	Urine, catheter	ESBL	–
	TS7670	Surgical wound		–
5633	EL3851	Rectum mucus	ESBL	–
5634	MD3414	Rectum mucus	ESBL	–
5635	UA15942	Urine	ESBL	–
	UA16057	Urine	ESBL	Turbid plaques
	UB15620	Urine	ESBL	–
5636	UB15822	Urine	ESBL	Turbid plaques

	UD15570	Urine	ESBL	–
	UE15406	Urine	ESBL	–
	EL3867	Rectum mucus	ESBL	Turbid plaques
5637	EL3870	Feces	ESBL	Turbid plaques
5638	EL3873	Rectum mucus	ESBL	–
5639	EL3875	Urine	ESBL	–

Table S2. Annotation of the predicted gene products of fHy-Eco03. The genes, gene location, gene size, and protein length are listed. LC-MS/MS analysis was performed to identify phage particle associated proteins (PPAPs). The best HHpred hits (with a probability above 50% and E-value below 1) are included [2]. A BLASTP search against the non-redundant protein sequences database (release update from February 6, 2021) was performed for every predicted gene product and the two results with the lowest E-values were recorded including query coverage, amino acid identity, and the accession numbers of the closest homologs. Note that due to the 3229 bp terminal repeats genes *g01* – *g11R* have two genomic locations. The fHy-Eco03 genome sequence with annotation has been deposited in the NCBI nucleotide database (GenBank) under accession number MW602648.

**fHy-Eco03 genome features**						**The best HHpred hits (Probability percentage/E-value)**	**BLASTP analysis**				
**Gene**	**Start position**	**End position**	**Gene size (bp)**	**Protein size (aa)**	**PPAP**		**The best hits**	**Query coverage %**	**E-value**	**Amino acid identity %**	**Accession number**
g01	114<br>**53426**	311<br>**53623**	198	65	no	No significant hits	hypothetical protein zoltan_21 [*Salmonella* phage zoltan]<br>hypothetical protein ciri_21 [*Salmonella* phage ciri]	93<br>93	0.007<br>0.007	40.32<br>40.32	QIN98827.1<br>QIN98756.1
g02	311<br>**53623**	736<br>**54048**	426	141	yes	1OB8_B, Holliday-junction resolvase {*Saccharolobus solfataricus*}(99.6%/1.3*10^{-14})	putative resolvase [*Salmonella* virus S144]<br>hypothetical protein HOV36_gp57 [*Salmonella* phage ZCSE2]	100<br>100	2e-77<br>1e-76	74.26<br>73.27	QMV47894.1<br>YP_009821772.1
g03R	962<br>**54274**	777<br>**54089**	186	61	no	No significant hits	hypothetical protein triss_20 [*Salmonella* phage triss]<br>hypothetical protein HWB04_gp70 [*Salmonella* phage UPF_BP2]	100<br>100	8e-34<br>2e-33	88.52<br>88.52	QIN99040.1<br>YP_009831725.1
g04R	1138<br>**54450**	959<br>**54271**	180	59	no	No significant hits	hypothetical protein BJD50_gp03 [*Salmonella* phage BP63]<br>hypothetical protein JEP7_56 [*Escherichia* phage JEP7]	100<br>100	7e-36<br>2e-35	98.31<br>98.31	YP_009302922.1<br>QOC55413.1
g05R	1383<br>**54695**	1138<br>**54450**	246	81	no	No significant hits	hypothetical protein HOV36_gp60 [*Salmonella* phage ZCSE2]<br>hypothetical protein HWC20_gp76 [*Salmonella* phage SE4]	100<br>92	2e-39<br>4e-39	77.78<br>82.67	YP_009821775.1<br>YP_009845656.1
g06R	1492<br>**54804**	1373<br>**54685**	120	39	no	No significant hits	hypothetical protein [*Salmonella* phage 8-19]<br>hypothetical protein HWC19_gp07 [*Salmonella* phage SE13]	94<br>97	1e-08<br>2e-08	67.57<br>65.79	QFR58182.1<br>YP_009845514.1
g07R	2122<br>**55434**	1550<br>**54862**	573	190	yes	No significant hits	hypothetical protein HOV36_gp67 [*Salmonella* phage ZCSE2]<br>hypothetical protein [*Salmonella* virus S144]	96<br>96	1e-74<br>3e-74	63.59<br>63.04	YP_009821782.1<br>QMV47903.1

g08R	2453 **55765**	2253 **55565**	201	66	no	No significant hits	hypothetical protein HOV36_gp63 [*Salmonella* phage ZCSE2]	98	4e-09	41.54	YP_009821778.1
							hypothetical protein [*Salmonella* virus S144]	98	5e-09	41.54	QMV47899.1
g09R	2684 **55996**	2457 **55769**	228	75	no	No significant hits	hypothetical protein brorfarstad_14 [*Salmonella* phage brorfarstad]	100	4e-30	66.67	QIN98305.1
							hypothetical protein vBSenM1_37 [*Salmonella* phage vB_SenM-1]	100	7e-30	66.67	QJQ40037.1
g10R	2930 **56242**	2754 **56066**	177	58	no	No significant hits	hypothetical protein [*Salmonella* virus S144]	100	1e-22	79.31	QMV47901.1
							hypothetical protein HWC20_gp71 [*Salmonella* phage SE4]	98	1e-22	82.46	YP_009845651.1
g11R	3918 **56412**	4262 **56242**	171	56	no	No significant hits	hypothetical protein HWD20_gp09 [*Salmonella* phage birk]	83	7e-04	38.30	YP_009857669.1
							hypothetical protein vBSalMLPSEYT_00001 [*Salmonella* phage vB_SalM-LPSEYT]	83	0.002	38.30	AWW14697.1
g12	4312	4461	495	164	yes	No significant hits	hypothetical protein nenneke_8 [*Salmonella* phage nenneke]	98	2e-23	40.00	QIN98440.1
							hypothetical protein rivia_8 [*Salmonella* phage rivia]	98	8e-23	38.79	EBY8042318.1
g13	4461	4697	237	78	no	No significant hits	No significant similarity found				
g14	4697	5368	672	223	yes	4PSS_A, Dihydrofolate reductase {*Escherichia coli*} (99.91%/$3.9*10^{-23}$)	dihydrofolate reductase [*Salmonella* phage vB_SalM-LPSEYT]	100	3e-92	59.82	AWW14768.1
							dihydrofolate reductase [*Salmonella* phage SE13]	100	1e-91	59.82	YP_009845524.1
g15	5365	5871	507	168	no	4MQB_B, Thymidylate kinase {*Staphylococcus aureus*} (99.71%/$5.5*10^{-15}$)	thymidylate kinase [*Salmonella* phage 8-19]	100	1e-98	80.95	QFR58193.1
							thymidylate kinase [*Salmonella* phage birk]	100	3e-98	80.95	YP_009857666.1
g16	5868	6590	723	240	yes	No significant hits	hypothetical protein HOV36_gp73 [*Salmonella* phage ZCSE2]	95	4e-15	30.80	YP_009821788.1
g17	6590	6880	291	96	no	No significant hits	hypothetical protein [*Salmonella* virus S144]	100	5e-37	59.38	QMV47832.1
							hypothetical protein HOV36_gp74 [*Salmonella* phage ZCSE2]	100	2e-36	58.33	YP_009821789.1
g18	7076	7399	324	107	no	No significant hits	hypothetical protein HWD20_gp03 [*Salmonella* phage birk]	87	3e-41	72.34	YP_009857663.1
							hypothetical protein HWC19_gp21 [*Salmonella* phage SE13]	87	3e-41	71.28	YP_009845528.1
	7389	8840	1452	483	yes		terminase large subunit [*Salmonella* phage SE4]	100	0.0	83.02	YP_009845640.1

g19						3CPE_A, Large terminase, gp17 {*Escherichia* virus T4} (100%/$3.5*10^{-32}$)	hypothetical protein HOV36_gp76 [*Salmonella* phage ZCSE2]	100	0.0	82.82	YP_009821791.1
g20	8842	10389	1548	515	yes	2JES_K, Portal protein {*Bacillus* phage SSP1} (99.96%/$1.1*10^{-26}$)	portal protein [*Salmonella* phage ciri]<br>portal protein [*Salmonella* phage BP63]	99<br>99	0.0<br>0.0	77.80<br>77.61	QIN98736.1<br>YP_009302940.1
g21	10566	11282	717	238	yes	21C6_A, Sin Nombre Virus Nucleocapsid Protein (94.9%/0.93)	hypothetical protein HOV36_gp78 [*Salmonella* phage ZCSE2]<br>scaffold protein [*Salmonella* phage SE4]	100<br>99	1e-109<br>2e-131	81.09<br>81.36	YP_009821793.1<br>YP_009845638.1
g22	11285	12253	969	322	yes	6R3A_G, Major capsid protein {*Bacillus* phage SSP1} (100%/$8.3*10^{-37}$)	major capsid protein [*Salmonella* phage brunost]<br>major capsid protein [*Salmonella* phage BP63]	100<br>83	0.0<br>0.0	87.38<br>87.38	QIN98734.1<br>YP_009302942.1
g23	12326	12694	369	122	no	5H0M_A, HNH endonuclease {*Geobacillus* virus E2} (99.17%/$3*10^{-11}$)	HNH endonuclease [*Salmonella* phage ZCSE2]<br>hypothetical protein [*Klebsiella* phage NL_ZS_3]	95<br>95	8e-50<br>1e-32	68.10<br>55.66	YP_009821717.1<br>QNN97356.1
g24	12730	13224	495	164	yes	5A21_D, Head-to-tail interface {*Bacillus* phage SSP1} (96.05%/0.27)	hypothetical protein HWC20_gp56 [*Salmonella* phage SE4]<br>putative head-to-tail connector complex 1 [*Salmonella* virus S144]	100<br>99	2e-79<br>4e-78	69.28<br>68.07	YP_009845636.1<br>QMV47838.1
g25	13226	13693	468	155	yes	No significant hits	putative head-to-tail connector complex 2 [*Salmonella* virus S144]<br>hypothetical protein HOV36_gp04 [*Salmonella* phage ZCSE2]	99<br>99	2e-52<br>3e-52	52.56<br>52.56	QMV47839.1<br>YP_009821719.1
g26	13695	14096	402	133	no	5A21_F, Head completion protein, gp16 {*Bacillus* phage SSP1} (97.96%/$4.0*10^{-4}$)	hypothetical protein HWC20_gp54 [*Salmonella* phage SE4]<br>hypothetical protein HWC19_gp28 [*Salmonella* phage SE13]	98<br>98	4e-52<br>2e-51	64.89<br>64.12	YP_009845634.1<br>YP_009845535.1
g27	14020	14553	534	177	yes	5A21_G, Tail-to-head joining protein, gp17 {*Bacillus* phage SSP1} (96.59%/0.075)	hypothetical protein BJD50_gp27 [*Salmonella* phage BP63]<br>hypothetical protein HWB04_gp48 [*Salmonella* phage UPF_BP2]	92<br>92	8e-60<br>2e-59	57.67<br>57.06	YP_009302946.1<br>YP_009831703.1
g28	14556	15176	621	206	yes	No significant hits	hypothetical protein HWD19_gp65 [*Salmonella* phage yarpen]<br>putative tail protein 1 [*Salmonella* virus S144]	97<br>99	9e-67<br>3e-64	54.23<br>54.63	YP_009857656.1<br>QMV47842.1
g29	15169	18132	2964	987	yes	No significant hits	putative tail protein 2 [*Salmonella* virus S144]<br>tail protein I [*Salmonella* phage ZCSE2]	98<br>98	0.0<br>0.0	78.32<br>78.22	QMV47843.1<br>YP_009821723.1

g30	18199	18630	432	143	yes	5NGJ_B, Tail tube protein {*Escherichia* phage T5} (95.2%/0.78)	hypothetical protein [*Pseudobutyrivibrio* sp.]	88	4e-16	44.09	MBE5909634.1
							Ig-like domain-containing protein [*Cloacibacillus porcorum*]	61	7e-16	55.06	WP_168950698.1
g31	18670	20787	2118	705	yes	4SHS_A, Hoc Head outer capsid protein {*Escherichia* phage RB69} (96.42%/0.98)	hypothetical protein [*Salmonella* phage 8-19]	100	0.0	66.71	QFR58208.1
							putative Ig-like domain-containing protein [*Salmonella* phage UPF_BP2]	100	0.0	66.85	YP_009831699.1
g32	20874	22007	1134	377	yes	3CPE_A, Tail sheath protein, gp18 {*Escherichia* virus T4} (98.6%/2.4*10^{-5})	hypothetical protein HWC19_gp34 [*Salmonella* phage SE13]	100	0.0	75.20	YP_009845541.1
							hypothetical protein brorfarstad_62 [*Salmonella* phage brorfarstad]	100	0.0	74.93	QIN98353.1
g33	22019	22450	432	143	yes	6GKX_, R-type bacteriocin tube protein CD1364 {*Clostridium difficile*} (97.56%/2.9*10^{-3})	hypothetical protein HOV36_gp12 [*Salmonella* phage ZCSE2]	99	1e-90	88.73	YP_009821727.1
							DUF3277 domain-containing protein [*Escherichia coli*]	99	2e-89	88.03	WP_166428470.1
g34	22469	22927	459	152	yes	No significant hits	hypothetical protein HOV36_gp13 [*Salmonella* phage ZCSE2]	97	2e-53	58.78	YP_009821728.1
							hypothetical protein HWC20_gp46 [*Salmonella* phage SE4]	97	2e-53	59.46	YP_009845626.1
g35	23078	24769	1692	563	yes	6V8I_AF, Tape measure protein, gp57 {*Staphylococcus* virus 80alpha} (97.7%/0.2)	hypothetical protein brorfarstad_59 [*Salmonella* phage brorfarstad]	100	0.0	60.84	QIN98350.1
							tail tape measure protein [*Salmonella* phage vB_SenM-1]	100	0.0	61.27	QJQ40070.1
g36	24766	25665	900	299	yes	No significant hits	hypothetical protein vBSenM1_71 [*Salmonella* phage vB_SenM-1]	99	3e-152	72.82	QJQ40071.1
							hypothetical protein HWD20_gp61 [*Salmonella* phage birk]	99	2e-151	72.48	YP_009857721.1
g37	25662	26054	393	130	yes	No significant hits	hypothetical protein HWB04_gp39 [*Salmonella* phage UPF_BP2]	100	2e-59	63.85	YP_009831694.1
							hypothetical protein BJD50_gp38 [*Salmonella* phage BP63]	100	3e-59	63.08	YP_009302957.1
g38	25996	26943	948	315	yes	1WRU_A, Baseplate gp44 {*Enterobacteria* phage Mu} (99.9%/1.7*10^{-21})	hypothetical protein vBSalMLPSEYT_00050 [*Salmonella* phage vB_SalM-LPSEYT]	98	3e-165	72.35	AWW14746.1
							hypothetical protein BJD50_gp39 [*Salmonella* phage BP63]	98	5e-165	72.35	YP_009302958.1
g39	26945	27586	642	213	yes		hypothetical protein HWD19_gp54 [*Salmonella* phage yarpen]	98	2e-122	78.10	YP_009857645.1

						4RU3_A, Cell puncturing protein gp41 {*Pseudomonas* phage SN} (99.9%/$1.1*10^{-26}$)	baseplate protein [*Salmonella* phage vB_SalM-LPSEYT]	98	2e-122	78.10	AWW14745.1
g40	27595	27954	360	119	no	5IW9_B, Baseplate wedge protein, gp25 {*Escherichia* virus T4} (99.72%/$1*10^{-15}$)	hypothetical protein HOV36_gp19 [*Salmonella* phage ZCSE2]	98	4e-65	99.25	YP_009821734.1
							hypothetical protein HWC20_gp40 [*Salmonella* phage SE4]	99	1e-64	98.49	YP_009845620.1
g41	27957	29114	1158	385	yes	5HX2_E, Baseplate wedge protein, gp6 {*Escherichia* virus T4} (100%/$3.5*10^{-30}$)	hypothetical protein brorfarstad_53 [*Salmonella* phage brorfarstad]	100	0.0	67.44	QIN98344.1
							hypothetical protein HWC19_gp43 [*Salmonella* phage SE13]	100	0.0	67.18	YP_009845550.1
g42	29107	29757	651	216	yes	No significant hits	hypothetical protein HOV36_gp21 [*Salmonella* phage ZCSE2]	100	7e-113	74.19	YP_009821736.1
							hypothetical protein vBSenM1_77 [*Salmonella* phage vB_SenM-1]	100	1e-110	73.27	QJQ40077.1
g43	29750	31099	1350	449	yes	5YVQ_A, Tail fiber protein S {*Enterobacteria* phage Mu} (96.59%/0.004)	putative tail fibers protein [*Salmonella* phage SE4]	100	0.0	67.26	YP_009845617.1
							tail fiber protein [*Salmonella* phage ZCSE2]	100	0.0	66.82	YP_009821737.1
g44	31096	31629	534	177	yes	5YVQ_B, Tail fiber assembly protein U {*Enterobacteria* phage Mu} (99.97%/$2.2*10^{-29}$)	hypothetical protein HOV36_gp23 [*Salmonella* phage ZCSE2]	98	3e-23	98.92	YP_009821738.1
							tail fibers protein [*Salmonella* phage SE4]	98	1e-20	96.24	YP_009845616.1
g45	31727	31972	246	81	yes	No significant hits	hypothetical protein HWC19_gp48 [*Salmonella* phage SE13]	96	9e-23	55.95	YP_009845555.1
							hypothetical protein vBSenM1_81 [*Salmonella* phage vB_SenM-1]	96	2e-22	55.95	QJQ40081.1
g46	31947	32510	564	187	yes	2ANV_A, Phage lysozyme {*Salmonella* phage P22} (99.81%/$6.6*10^{-18}$)	putative endolysin [*Salmonella* virus S144]	97	5e-122	90.71	QMV47861.1
							lysozyme [*Salmonella* phage vB_SalM-LPSEYT]	97	1e-120	90.71	AWW14736.1
g47	32480	32830	351	116	yes	4LIN_G, Tail needle protein, gp26 {*Salmonella* phage P22} (95.71%/0.5)	putative IM-spanin [*Salmonella* virus S144]	100	6e-37	62.07	QMV47862.1
							hypothetical protein HOV36_gp27 [*Salmonella* phage ZCSE2]	100	7e-37	62.07	YP_009821742.1
g48	32814	33029	216	71	no	No significant hits	hypothetical protein rivia_44 [*Salmonella* phage rivia]	94	1e-29	74.63	QIN98547.1
							hypothetical protein HWD20_gp48 [*Salmonella* phage birk]	94	7e-28	73.13	YP_009857708.1

g49R	33320	333042	279	92	yes	No significant hits	hypothetical protein HWC19_gp52 [*Salmonella* phage SE13]	98	2e-54	89.01	YP_009845559.1
							ryanodine receptor [*Salmonella* phage vB_SalM-LPSEYT]	98	1e-52	85.71	AWW14733.1
g50R	33544	33320	225	74	no	4IJJ_B, C4-type zinc finger protein, DksA/TraR family {*Pseudomonas aeruginosa*}(99.7%/4.7*10^{-16})	hypothetical protein HWC19_gp53 [*Salmonella* phage SE13]	100	5e-42	89.19	YP_009845560.1
							DnaK suppressor protein [*Salmonella* phage vB_SalM-LPSEYT]	100	2e-40	85.14	AWW14732.1
g51R	33692	33537	156	51	no	No significant hits	No significant similarity found				
g52R	33964	33689	276	91	no	No significant hits	hypothetical protein HOV36_gp29 [*Salmonella* phage ZCSE2]	100	4e-49	99.51	YP_009821744.1
							hypothetical protein HWC19_gp54 [*Salmonella* phage SE13]	100	3e-41	99.35	YP_009845561.1
g53R	35953	33986	1968	655	yes	4X0Q_A, DNA polymerase theta {*Homo sapiens*}(100%/3.5*10^{-70})	DNA polymerase [*Salmonella* phage vB_SenM_PA13076]	100	0.0	85.50	ATI16200.1
							DNA polymerase [*Salmonella* phage BP63]	100	0.0	85.50	YP_009302972.1
g53.1R	35916	35458	459	152	yes	No significant hits	No significant similarity found				
g54R	36947	35955	993	330	no	4TR6_B, DNA polymerase III subunit beta {*Bacillus subtilis*}(100%/8.9*10^{-43})	DNA polymerase beta subunit [*Salmonella* phage LSE7621]	99	4e-166	66.87	QBJ00954.1
							DNA polymerase beta subunit [*Salmonella* phage vB_SenM_PA13076]	99	8e-166	66.57	ATI16199.1
g55R	37912	36983	930	309	yes	4EZ8_A, Thymidylate synthase {*Mus musculus*}(100%/1*10^{-58})	thymidylate synthase [*Salmonella* phage ZCSE2]	100	5e-132	60.97	YP_009821747.1
							thymidylate synthase [*Salmonella* phage SE4]	100	2e-128	59.03	YP_009845606.1
g56R	38514	37912	603	200	yes	3NL9_A, NTP pyro-phosphohydrolase {*Exiguobacterium sibiricum*}(99.96%/6.9*10^{-29})	hypothetical protein HWC20_gp25 [*Salmonella* phage SE4]	99	4e-91	65.66	YP_009845605.1
							hypothetical protein HOV36_gp33 [*Salmonella* phage ZCSE2]	99	4e-90	64.14	YP_009821748.1
g57R	38899	38501	399	132	yes	No significant hits	hypothetical protein HWB04_gp21 [*Salmonella* phage UPF_BP2]	100	4e-25	42.54	YP_009831676.1
							hypothetical protein PA13076_52 [*Salmonella* phage vB_SenM_PA13076]	100	6e-25	42.54	ATI16196.1
g58	38888	39034	147	48	no	No significant hits	No significant similarity found				
g59R	39379	39254	126	41	no	No significant hits	No significant similarity found				
g60R	39964	39488	477	158	yes	No significant hits	hypothetical protein HWC19_gp60 [*Salmonella* phage SE13]	82	5e-68	91.60	YP_009845567.1

							hypothetical protein HOV36_gp36 [*Salmonella* phage ZCSE2]	97	9e-60	81.82	YP_009821751.1
g61R	40290	40075	216	71	no	No significant hits	hypothetical protein HOV36_gp37 [*Salmonella* phage ZCSE2]	100	2e-19	50.70	YP_009821752.1
							hypothetical protein HWC20_gp21 [*Salmonella* phage SE4]	100	4e-19	50.70	YP_009845601.1
g62R	41941	40268	1674	557	yes	2OCA_A, ATP-dependent helicase, uvsW {*Escherichia* virus T4} (100%/$2.6*10^{-33}$)	ATP-dependent RNA helicase [*Salmonella* phage ZCSE2]	99	0.0	84.41	YP_009821753.1
							putative ATP-dependent helicase [*Salmonella* virus S144]	99	0.0	84.23	QMV47876.1
g63R	42877	42005	873	290	yes	3SM4_C, Exonuclease {*Escherichia* virus Lambda} (100%/$5.3*10^{-11}$)	hypothetical protein HWC20_gp19 [*Salmonella* phage SE4]	98	9e-147	69.79	YP_009845599.1
							hypothetical protein HOV36_gp40 [*Salmonella* phage ZCSE2]	98	5e-144	68.40	YP_009821755.1
g64R	43388	42840	549	182	no	1U3E_M, HNH homing endonuclease {*Bacillus* virus SPO1} (93.91%/$1.6*10^{-12}$)	HNH endonuclease [*Acidibacillus sulfuroxidans*]	92	2e-25	41.14	WP_109431645.1
							homing endonuclease [*Enterobacteria* phage JenK1]	92	4e-25	41.14	YP_009219324.1
g65R	43806	43357	450	149	no	2W4L_F, deoxycytidylate deaminase {*Homo sapiens*} (99.93%/$4.4*10^{-24}$)	hypothetical protein HOV36_gp41 [*Salmonella* phage ZCSE2]	91	6e-41	52.48	YP_009821756.1
							deoxycytidylate deaminase [*Salmonella* phage birk]	91	3e-40	49.29	YP_009857696.1
g66R	444393	43866	528	175	yes	No significant hits	hypothetical protein HWD20_gp35 [*Salmonella* phage birk]	100	2e-88	71.82	YP_009857695.1
							hypothetical protein brorfarstad_33 [*Salmonella* phage brorfarstad]	100	2e-87	69.61	QIN98324.1
g67R	45388	44495	894	297	yes	1C9K_C, Adenosyl-cobinamide kinase {*Salmonella typhimurium*} (99.33%/$1.9*10^{-11}$)	putative P-loop containing nucleoside triphosphatehydrolase [*Salmonella* virus S144]	93	8e-162	76.70	QMV47880.1
							hypothetical protein HWC20_gp16 [*Salmonella* phage SE4]	93	1e-161	77.14	YP_009845596.1
g68R	45619	45389	231	76	no	No significant hits	hypothetical protein HWC20_gp15 [*Salmonella* phage SE4]	100	8e-23	57.89	YP_009845595.1
							hypothetical protein [*Salmonella* virus S144]	100	1e-22	59.21	QMV47881.1
g69R	46099	45677	423	140	no	No significant hits	hypothetical protein HWC20_gp14 [*Salmonella* phage SE4]	95	2e-19	36.17	YP_009845594.1

							hypothetical protein HOV36_gp45 [*Salmonella* phage ZCSE2]	94	4e-19	36.69	YP_009821760.1
g70R	47200	46148	1053	350	yes	No significant hits	hypothetical protein HOV36_gp46 [*Salmonella* phage ZCSE2]	100	5e-97	47.21	YP_009821761.1
							hypothetical protein [*Salmonella* virus S144]	100	4e-96	46.93	QMV47883.1
g71R	47572	47273	300	99	no	No significant hits	hypothetical protein HWC20_gp12 [*Salmonella* phage SE4]	95	1e-35	61.05	YP_009845592.1
							hypothetical protein HOV36_gp47 [*Salmonella* phage ZCSE2]	96	1e-35	60.42	YP_009821762.1
g72R	47835	47569	267	88	no	6QX5_G, Portal protein {*Escherichia* phage T7} (91.71%/0.31)	hypothetical protein HWD19_gp25 [*Salmonella* phage yarpen]	97	3e-11	40.22	YP_009857616.1
							hypothetical protein triss_27 [*Salmonella* phage triss]	97	2e-10	40.22	QIN99047.1
g73R	48157	47798	360	119	no	No significant hits	No significant similarity found				
g74R	48593	48144	450	149	yes	No significant hits	hypothetical protein BJD50_gp72 [*Salmonella* phage BP63]	95	9e-69	71.83	YP_009302991.1
							hypothetical protein vBSenM1_22 [*Salmonella* phage vB_SenM-1]	95	9e-69	70.83	QJQ40022.1
g75R	48843	48568	276	91	yes	No significant hits	No significant similarity found				
g76R	49084	48845	240	79	no	No significant hits	hypothetical protein HWC20_gp06 [*Salmonella* phage SE4]	100	8e-39	77.22	YP_009845586.1
							hypothetical protein HOV36_gp52 [*Salmonella* phage ZCSE2]	88	3e-32	71.43	YP_009821767.1
g77R	49534	49175	360	119	no	No significant hits	hypothetical protein HWC20_gp05 [*Salmonella* phage SE4]	100	4e-65	80.67	YP_009845585.1
							hypothetical protein [*Salmonella* virus S144]	100	1e-31	53.60	QMV47891.1
g78R	49956	49531	426	141	yes	No significant hits	hypothetical protein HWC20_gp04 [*Salmonella* phage SE4]	100	9e-56	58.87	YP_009845584.1
							hypothetical protein HOV36_gp54 [*Salmonella* phage ZCSE2]	100	2e-55	58.16	YP_009821769.1
g79	50114	50443	330	109	yes	No significant hits	hypothetical protein emhyr_24 [*Salmonella* phage emhyr]	97	6e-49	72.64	QIN98901.1
							glutaredoxin [*Salmonella* phage ZCSE2]	97	9e-47	70.75	YP_009821770.1
g80	50421	52952	2532	843	yes	6A9W_A, Primase {*Nitratiruptor* phage NrS-1} (99.88%/$8.9*10^{-21}$)	DNA primase/helicase [*Salmonella* phage SE13]	100	0.0	79.15	YP_009845509.1
							DNA primase/helicase [*Salmonella* phage vB_SenM-1]	100	0.0	78.91	QJQ40027.1

Table S3. Primers to amplify control genes encoding toxic and non-toxic gene products.

Phage	Gene	Primer	Sequence (5' – 3')	Restriction site	Size
Toxic gene products					
fHy-Kpn01	*g10*	Gp10FW	GCAGCGGCCGCATGATTAAGTACGATGTATACAAG	*Not*I	210
		Gp10RV	GGTCCATGGCTACTGTGAGCATAGGCTG	*Nco*I	
	g22	Gp22FW	GCAGCGGCCGCATGATTGACAGAGAAGAGATAC	*Not*I	549
		Gp22RV	GGTCCATGGTTAATATGCATCACGCACC	*Nco*I	
	g38	Gp38FW	GCAGCGGCCGCATGAAGTTAAAACACACTAGTAA	*Not*I	441
		Gp38RV	GGTCCATGGTCATTCTGACCTCACTAAATG	*Nco*I	
ϕR1-RT	*g137*	Gp137FW	GCAGCGGCCGCATGAAAATTGCTGAACTAAT	*Not*I	183
		Gp137RV	GGTCCATGGCTATGAGGACTTAGAAATTGT	*Nco*I	
T4	*regB*	RegBFW	GATCGCGGCCGCCCATGACTATCAATACAGAAG	*Not*I	491
		RegBRV	GGCCGCTAGCTTACCTCATTGAGTTTTAATTAC	*Nhe*I	
Non-toxic gene products					
ϕR1-RT	*g119*	Gp119FW	GCAGCGGCCGCATGAAAACGTATAAAGAATTTTTG	*Not*I	591
		Gp119RV	GGTCCATGGTTAACGAACGTTAGTGCCA	*Nco*I	
	g121	Gp121FW	GCAGCGGCCGCATGAAAACCTATAATGAATTTATC	*Not*I	273
		Gp121RV	GGTCCATGGTTAGGAAGCTTTTTTAAGC	*Nco*I	
	g150	Gp150FW	GCAGCGGCCGCATGATTAAAGTTAATGAGC	*Not*I	1728
		Gp150RV	GGTGCTAGCCTATCCAATATCAATTCGTGAA	*Nhe*I	
	g178	Gp178FW	GCAGCGGCCGCATGAGCAATATTAACCAGC	*Not*I	1275
		Gp178RV	GGTCCATGGTTATCCTGCTATTAGTTTAGG	*Nco*I	
	g246	Gp246FW	GCAGCGGCCGCATGTCTTTAAATGAAATG	*Not*I	939
		Gp246RV	GGTCCATGGTTAAAAATCATTTGCATG	*Nco*I	

Table S4. Primers used to amplify the HPUF genes of fHy-Eco03. Sequences are presented in 5´to 3´direction and restriction sites (*Nco*I, *Nhe*I) are underlined. Sizes of the genes in basepairs (bp) are given including the start and the stop codons.

Gene	Forward primer	Reverse primer	Size (bp)
g01	GCA<u>GCGGCCGC</u>ATGAGCTATACTGACCAACA	GGT<u>CCATGG</u>TTACAGTTGACCTCTCAGG	198
g03	GCA<u>GCGGCCGC</u>GTGATTGGCTTAATCCTGG	GGT<u>CCATGG</u>TTAGTCTTGTTTCAACTCATCG	186
g04	GCA<u>GCGGCCGC</u>ATGTTTAAATTCTTACAACGTAACC	GGT<u>CCATGG</u>TCACAAGTAATCTCCTTCAAC	180
g05	GCA<u>GCGGCCGC</u>ATGTTTTCTGAGGAGCAACT	GGT<u>CCATGG</u>TTATTTGCTCCTTACTCCAAGT	246
g06	GCA<u>GCGGCCGC</u>ATGAAAAAGCATGTTGTTGAG	GGT<u>CCATGG</u>TCAGAAAACATAAGACAACACC	120
g08	GCA<u>GCGGCCGC</u>ATGCCATTAATCAAAGTTACAG	GGT<u>CCATGG</u>TCAAGAAATAATTACCTCAGTAAC	201
g09	GCA<u>GCGGCCGC</u>ATGGTAACATTCACAACTATCC	GGT<u>CCATGG</u>TTAAACCTCAAATTTTGATTCGTC	228
g10	GCA<u>GCGGCCGC</u>ATGATGAAAGAATTGACATTGAC	GGT<u>CCATGG</u>CTATTGTAACTTTGTACTAATTTGA	177
g11	GCA<u>GCGGCCGC</u>ATGCATACTCAATTAATCATCTGG	GGT<u>CCATGG</u>TCATTCTCCTGAGTTAAATTGTG	171
g13	GCA<u>GCGGCCGC</u>ATGACTGTAGTTGCACCT	GGT<u>CCATGG</u>TCATTTTACCTTAAATTTAAGCCA	237
g15	GCA<u>GCGGCCGC</u>ATGAACATCATAGTTGAGGGA	GGT<u>CCATGG</u>TCATTTAGTCCACTCCTTATCG	507
g17	GCA<u>GCGGCCGC</u>ATGGACTTATTCGACTTAGTTGA	GGT<u>CCATGG</u>TTATGCCATTTCTACCAGCA	291
g18	GCA<u>GCGGCCGC</u>ATGGCACGAAAACCACAA	GGT<u>CCATGG</u>TCAAGATTCATTATTATCCTCGTCC	324
g23	GCA<u>GCGGCCGC</u>ATGGGTGGCGCAAGAATC	GGT<u>CCATGG</u>CTAATTTTGTACAAGTAAACCAGAG	369
g26	GCA<u>GCGGCCGC</u>ATGGGCCTGTTGAAGATTAC	GGT<u>CCATGG</u>TCAGGTAATTGTAAAAGCCGG	402

g40	GCAGCGGCCGCATGAGAACCAGACGACTTGA	GGTCCATGGCTACCCCGCAACAGTGTC	360
g48	GCAGCGGCCGCTTGACCTCATGCAGTAAAAAG	GGTCCATGGTTAATTGGCCCCTTCACT	216
g50	GCAGCGGCCGCATGATTGAATCTACACGCAC	GGTCCATGGTTAGCGCTTTCCTTTCTTTT	225
g51	GCAGCGGCCGCATGAGAAAGTTTACCCTGGA	GGTCCATGGTCAATCATATTTCTTAGCCAAGT	156
g52	GCAGCGGCCGCGTGTCTAATATAGGTTTAGAGTACC	GGTCCATGGTCATTTGCATTCTTCCTCG	276
g58	GCAGCGGCCGCTTGGATATTCATGCTTCGCTC	GGTCCATGGTCATGCAACACCATCCTTGTT	147
g59	GCAGCGGCCGCTTGAATGTAGCATTAAGTGTGGC	GGTCCATGGTCAGCGAGCAGACTCGAAC	126
g61	GCAGCGGCCGCTTGGGCAACCGAGAACAG	GGTCCATGGCTAAAAAGGCACTTTATAGTCAAAAG	216
g64	GCAGCGGCCGCATGTTTGTGCCGATTCCG	GGTCCATGGTCAATTCGCTTCTTCAGCG	549
g65	GCAGCGGCCGCATGCAGAACCTTTGGGATA	GGTCCATGGTCAGCTTGTGTAGCCGG	450
g68	GCAGCGGCCGCATGTATACGTTCAATCAATTCAAA	GGTCCATGGTTAATACCCCTCTGTTAACATC	231
g69	GCAGCGGCCGCATGGAAGCCACCAAGTCT	GGTCCATGGTTATTTATACACTTCAATGGTACAC	423
g71	GCAGCGGCCGCATGAGTGAGAACTTCGAGAAG	GGTCCATGGTTATTTTAGTGCCCCTTCGCA	300
g72	GCAGCGGCCGCATGAAAGCGGTCAACAAGAA	GGTCCATGGTCATGGCAGTTCTACCTGTAC	267
g73	GCAGCGGCCGCATGGCGACGAGTAAAAACG	GGTCCATGGTTATCTTCCTGACTGTACTTCTTG	360
g76	GCAGCGGCCGCATGAAACATCAGGGCACA	GGTCCATGGTTATCTCCAGCGTGCATG	240
g77	GCAGCGGCCGCATGAAAACAATGTTTGTATACAAG	GGTCCATGGTCACAGGCCTAACACCTC	360

Table S5. Workflow for the NGS assay bioinformatics. The raw NGS read data was analyzed using the Puhti computer environment at CSC (the Finnish Centre for Scientific Computing) following the protocol outlined below.

Step	Description	Command or script
1	The sequences over the ligation joints in the predicted plasmids on both sides of the HPUF gene fragment were determined *in silico* as described for table S8 and illustrated in figure 1b. A text document containing each sequence on its own line was prepared and saved under the name **list.txt**. This file was uploaded by WinSCP to the Puhti directory containing the NGS raw data files.	
2	Bio tools were activated using the commands	$ module load biokit<br>and<br>$ module load velvet
3	The compressed fastq.gz NGS sequence read files were uncompressed with the gunzip command	$ gunzip file_name.gz
4	The paired end fastq-files were interleaved to a single file using the shufflesequences command.	$ shuffleSequences_fastq.pl **read_file_1.fq read_file_2.fq file_name.fastq**
5	Alignments were run as a batch job by first editing the bash script text file template (file_name.sh) to include the required file names and paths	#!/bin/bash -l<br>#SBATCH -o std1.out<br>#SBATCH -e std1.err<br>#SBATCH -p small<br>#SBATCH --account=skurnik<br>#SBATCH --ntasks=1<br>#SBATCH --cpus-per-task=1<br>#SBATCH --nodes=1<br>#SBATCH -t 48:00:00<br>#SBATCH --mem=128000

```
module load biokit
#change directory to the one where you
have the data
cd /file_path

# insert the file name for your sequence
segment text file
a=1
for pat in $(cat
sequencelist_file_name.txt)
do
#insert the fastq filename of your
interleaved paired end reads
    fuzznuc -pattern "$pat"
file_name.fastq -rformat excel -filter |
awk '{ if ( $1 != "SeqName") print $1}' |
sort | uniq > name_$a
    (( a = a + 1))
done
```

6	The bash file can be edited using Nano (version 2.3.1) with the nano command	$ nano **file_name.sh**
7	To save the changes and to exit Nano the CTRL+O and CTRL+X commands were used	
8	The batch job was submitted by using the sbatch command	$ sbatch **file_name.sh**
9	The batch job results in a number of files with a name containing a running number corresponding to each sequence line in the **list.txt** file created in point 1. Each name_N file created contains the information of the number of reads containing the searched sequence	

Table S6. LC-MS/MS analysis of the proteome of the fHy-Eco03 virion particles with the hits sorted by their decreasing score values. Note that the values in last three columns represent the encoded amino acids within the full open reading frame inside which the predicted gene resides.

Gene product	Predicted function of the gene product	Gene location	Score	Coverage	# Peptides	# PSMs	# AAs	MW [kDa]	calc. pI
Gp35	Tape measure protein	23078..24769	289,05	60,35	29	79	565	61,2	9,06
Gp29	No significant hits	15169..18132	228,44	52,48	40	66	987	111,5	5,33
Gp22	Major capsid protein	11285..12253	206,47	70,06	18	53	324	34,6	5,07
Gp30	Tail tube protein	18199..18630	144,07	85,71	14	47	154	16,4	5,02
Gp41	Baseplate wedge protein	27957..29114	168,29	59,08	19	46	391	42,6	4,77
Gp36	No significant hits	24766..25665	112,65	22,67	6	37	300	32,5	5,07
Gp70	No significant hits	(46148..47200)c	112,32	53,52	17	35	355	40,1	8,65
Gp80	DNA primase / helicase	50421..52952	90,97	36,57	23	30	845	96,4	6,61
Gp67	Putative NTP hydrolase	(44495..45388)c	76,39	41,75	12	23	297	33,6	5,88
Gp28	No significant hits	14556..15176	79,88	68,57	8	20	210	21,8	5,15
Gp27	Tail-to-head joining protein	14020..14553	56,33	42,50	10	19	200	22,5	6,54
Gp55	Thymidylate synthase	(36983..37912)c	67,18	58,59	11	18	326	36,9	5,82
Gp31	Outer capsid protein	18670..20787	42,43	14,41	11	17	708	73,8	4,40
Gp43	Tail fiber protein	29750..31099	56,48	50,78	12	17	451	48,6	6,89
Gp20	Portal protein	8842..10389	58,74	40,61	10	16	522	58,6	5,03
Gp32	Tail sheath protein	20874..22007	48,90	32,22	9	14	388	42,8	5,10
Gp37	No significant hits	25662..26054	46,54	55,15	5	14	136	14,9	4,88
Gp14	Dihydrofolate reductase	4697..5368	37,98	47,79	7	12	226	25,4	6,40
Gp39	No significant hits	26945..27586	47,75	44,44	5	12	216	23,1	4,84
Gp62	ATP dependent helicase	(40268..41941)c	37,36	27,03	9	12	566	63,0	8,51
Gp21	Scaffold protein	10566..11282	45,45	33,75	4	11	240	26,8	6,20
Gp16	No significant hits	5868..6590	32,98	35,42	5	8	240	27,1	5,64
Gp19	Large terminase subunit	7389..8840	25,81	22,94	6	8	497	56,2	7,39

Gp25	No significant hits	13226..13693	21,57	35,54	5	8	166	19,0	10,18
Gp60	No significant hits	(40268..41941)c	29,40	44,91	6	8	167	18,1	8,48
Gp12	No significant hits	3967..4461	23,45	46,95	3	7	164	19,1	5,07
Gp33	DUF3277 domain-containing protein	22019..22450	18,90	43,75	6	7	144	15,8	5,22
Gp38	Baseplate protein	25996..26943	24,50	24,07	5	7	324	35,7	8,19
Gp53	DNA polymerase	(33986..35953)c	25,84	13,22	5	7	658	73,5	6,70
Gp42	No significant hits	29107..29757	22,45	33,49	5	6	218	24,5	6,18
Gp66	No significant hits	(43866..44393)c	15,56	46,93	3	6	179	19,0	5,05
Gp34	No significant hits	22469..22927	20,70	24,20	3	5	157	16,7	6,57
Gp56	NTP hydrolase	(38501..38899)c	16,88	21,95	4	5	205	23,6	5,25
Gp74	No significant hits	(48144..48593)c	13,78	31,33	3	5	150	16,8	8,90
Gp24	Head-to-tail connector	12730..13224	12,48	26,49	3	4	185	20,8	5,74
Gp45	No significant hits	31727..31972	12,06	12,06	2	4	141	15,6	8,94
Gp63	Exonuclease	(42005..42877)c	13,99	16,06	3	4	249	29,0	7,43
Gp57	No significant hits	(38501..38899)c	11,22	23,60	3	4	161	19,3	9,03
Gp53.1	No significant hits	(35458..35916)c	12,27	30,67	4	4	163	18,3	11,17
Gp78	No significant hits	(49531..49956)c	16,04	28,48	2	4	151	17,4	5,34
Gp75	No significant hits	(48568..48843)c	12,12	60,38	3	4	106	12,2	4,88
Gp79	No significant hits	50114..50443	6,22	28,80	3	3	125	14,3	9,26
Gp02	Holliday-junction resolvase	311..736	3,32	21,97	2	2	173	19,5	8,62
Gp44	Til fiber assembly protein	31096..31629	5,71	16,13	2	2	186	20,6	5,08
Gp46	Lysozyme	31947..32510	3,83	16,75	2	2	191	21,2	9,00
Gp47	Spanin	32480..32830	7,81	18,75	2	2	128	13,9	8,98
Gp49	No significant hits	(33042..33320)c	6,10	17,17	2	2	99	11,0	7,28
Gp07	No significant hits	(1550..2122)c	4,31	12,32	2	2	203	22,5	5,27

Table S7. Results from plating assay of control genes.

Type of gene	Gene	Experiment 1			Experiment 2			Average	SD
		CFU1	CFU2	CFU3	CFU1	CFU2	CFU3		
Toxic	*g137*	85	103	125	114	122	104	109	13
	g38	65	57	93	78	54	77	71	13
	g22	16	17	16	12	10	19	15	3
	g10	8	12	15	4	5	8	9	4
	regB	4	5	5	8	7	12	7	3
Non-toxic	*g178*	760	624	788	451	391	447	577	157
	g119	350	401	352	742	797	794	573	206
	g121	366	335	320	375	378	312	348	26
	g246	247	278	246	350	376	332	305	51
	g150	199	200	189	356	312	376	272	78

Table S8. Results of the NGS assay with control genes. For each gene, two pairs of complementary sequences over the ligation joints were determined *in silico* containing 15-25 nt of the gene and plasmid specific sequences (the latter underlined). The joints named as *gx_atg* and *gx_stop* are sequences specific for the forward strand containing the start or stop codons of the inserted genes, while sequences named *gx_atgrev* and *gx_stoprev* are the corresponding complementary sequences from the reverse strand. Elp1 and Elp2 stand for plasmid DNA isolated from replicate transformations. The reads containing these joint sequences were extracted from the NGS sequence read files as described in Table S6. The joint-read totals for each gene and their percentages of total joint-reads were calculated. These were then used to calculate the plasmid DNA ligation mixture abundance ratios.

Gene	Joint	Joint sequence	Pooled ligation mixture			Pooled plasmid DNA				Plasmid/ligation ratio
			N joint-reads	Gene total	Gene %	*N* joint-reads Elp1	*N* joint-reads Elp2	Gene total	Gene %	
g10	*g10*_atg	CCAAGCTTGCATGCCTGCAGGCGGCCGCATGATTAAGTACGATGT	36			11	29			
	*g10*_atgrev	TATACATCGTACTTAATCATGCGGCCGCCTGCAGGCATGCAAGCT	3			10	13			
	*g10*_stop	CAGCCTATGCTCACAGTAGCCATGGAGCTAGCTCTAGAGGATCCC	16			148	124			
	*g10*_stoprev	GGGATCCTCTAGAGCTAGCTCCATGGCTACTGTGAGCATAGGCTG	53	108	0,86	152	143	630	0,13	0,15
g22	*g22*_atg	CCAAGCTTGCATGCCTGCAGGCGGCCGCATGATTGACAGAGAAGA	193			22	13			
	*g22*_atgrev	TCTCTTCTCTGTCAATCATGCGGCCGCCTGCAGGCATGCAAGCTT	0			32	7			
	*g22*_stop	GGTGCGTGATGCATATTAACCATGGAGCTAGCTCTAGAGGATCCC	5			40	94			
	*g22*_stoprev	GGGATCCTCTAGAGCTAGCTCCATGGTTAATATGCATCACGCACC	193	391	3,10	21	94	323	0,07	0,02
g38	*g38*_atg	CCAAGCTTGCATGCCTGCAGGCGGCCGCATGAAGTTAAAACACACTAG	1229			17191	16249			
	*g38*_atgrev	TTACTAGTGTGTTTTAACTTCATGCGGCCGCCTGCAGGCATGCAAGCT	3			14108	13946			
	*g38*_stop	GGGATCCTCTAGAGCTAGCTCCATGGTCATTCTGACCTCACTAAATG	614			17730	16471			
	*g38*_stoprev	CATTTAGTGAGGTCAGAATGACCATGGAGCTAGCTCTAGAGGATCCC	23	1869	14,84	17760	16749	130204	26,83	1,81
g119	*g119*_atg	CCAAGCTTGCATGCCTGCAGGCGGCCGCATGAAAACGTATAAAGAATT	877			11986	11896			
	*g119*_atgrev	AATTCTTTATACGTTTTCATGCGGCCGCCTGCAGGCATGCAAGCTTGG	4			11539	11460			
	*g119*_stop	TGGCACTAACGTTCGTTAACCATGGAGCTAGCTCTAGAGGATCCC	26			12216	12346			
	*g119*_stoprev	GGGATCCTCTAGAGCTAGCTCCATGGTTAACGAACGTTAGTGCCA	567	1474	11,70	11503	11787	94733	19,52	1,67
g121	*g121*_atg	CCAAGCTTGCATGCCTGCAGGCGGCCGCATGAAAACCTATAATGAATT	156			9467	9211			
	*g121*_atgrev	AATTCATTATAGGTTTTCATGCGGCCGCCTGCAGGCATGCAAGCTTGG	2			10055	10311			
	*g121*_stop	GCTTAAAAAAGCTTCCTAACCATGGAGCTAGCTCTAGAGGATCCC	24			9823	9688			
	*g121*_stoprev	GGGATCCTCTAGAGCTAGCTCCATGGTTAGGAAGCTTTTTTAAGC	170	352	2,79	9087	8714	76356	15,74	5,63
g137	*g137*_atg	CCAAGCTTGCATGCCTGCAGGCGGCCGCATGAAAATTGCTGAACTAAT	16			7000	7362			
	*g137*_atgrev	ATTAGTTCAGCAATTTTCATGCGGCCGCCTGCAGGCATGCAAGCTTGG	0			8356	8866			

	*g137*_stop	ACAATTTCTAAGTCCTCATAGCCATGGAGCTAGCTCTAGAGGATCCC	15			9320	9287			
	*g137*_stoprev	GGGATCCTCTAGAGCTAGCTCCATGGCTATGAGGACTTAGAAATTGT	30	61	0,48	6716	6924	63831	13,15	27,17
g150	*g150*_atg	CCAAGCTTGCATGCCTGCAGGCGGCCGCATGATTAAAGTTAATGAGC	1641			2398	2625			
	*g150*_atgrev	GCTCATTAACTTTAATCATGCGGCCGCCTGCAGGCATGCAAGCTTGG	3			2320	2489			
	*g150*_stop	CACGAATTGATATTGGATAGGCTAGCTCTAGAGGATCCCCGGGTAC	17			2162	2245			
	*g150*_stoprev	GTACCCGGGGATCCTCTAGAGCTAGCCTATCCAATATCAATTCGTG	1032	2693	21,38	1950	2288	18477	3,81	0,18
g178	*g178*_atg	CCAAGCTTGCATGCCTGCAGGCGGCCGCATGAGCAATATTAACCAGC	1387			7335	6952			
	*g178*_atgrev	GCTGGTTAATATTGCTCATGCGGCCGCCTGCAGGCATGCAAGCTTGG	0			6305	6202			
	*g178*_stop	AAACTAATAGCAGGATAACCATGGAGCTAGCTCTAGAGGATCCC	9			6111	5619			
	*g178*_stoprev	GGGATCCTCTAGAGCTAGCTCCATGGTTATCCTGCTATTAGTTT	1342	2738	21,74	6462	6341	51327	10,58	0,49
g246	*g246*_atg	CCAAGCTTGCATGCCTGCAGGCGGCCGCATGTCTTTAAATGAAATG	1487			6475	6464			
	*g246*_atgrev	CATTTCATTTAAAGACATGCGGCCGCCTGCAGGCATGCAAGCTTGG	9			6219	6227			
	*g246*_stop	CATGCAAATGATTTTTAACCATGGAGCTAGCTCTAGAGGATCCC	19			6789	6478			
	*g246*_stoprev	GGGATCCTCTAGAGCTAGCTCCATGGTTAAAAATCATTTGCATG	1396	2911	23,11	5442	5254	49348	10,17	0,44
regB	*regB*_atg	CCAAGCTTGCATGCCTGCAGGCGGCCGCATGACTATCAATACAG	0			0	0			
	*regB*_atgrev	CTGTATTGATAGTCATGCGGCCGCCTGCAGGCATGCAAGCTTGG	0			0	0			
	*regB*_stop	ATTAAAACTCAATGAGGTAAGGCTAGCTCTAGAGGATCCCC	0			0	0			
	*regB*_stoprev	GGGGATCCTCTAGAGCTAGCCTTACCTCATTGAGTTTTAAT	0	0	0,00	0	0	0	0,00	
		**Total reads**		**12597**	**100%**			**485229**	**100%**	

Table S9. Plating assay results of 23 HPUF-encoding genes of fHy-Eco03.

	Control genes					Ligation 1					Ligation 2					Fraction of *g178*			
	CFU1	CFU2	CFU3	Average	SD	CFU1	CFU2	CFU3	Average	SD	CFU1	CFU2	CFU3	Average	SD	Lig 1	Lig 2	Average	SD
regB	11	9	17	12	3														
g178	391	451	447	430	27														
g01						353	321	419	364	41	186	303	175	221	58	0.847	0.515	0.681	0.166
g03						448	446	419	438	13	565	362	349	425	99	1.018	0.989	1.003	0.014
g04						263	312	247	274	28	278	324	311	304	19	0.637	0.708	0.672	0.035
g05						47	80	81	69	16	81	83	52	72	14	0.161	0.167	0.164	0.003
g06						400	389	343	377	25	225	222	185	211	18	0.878	0.49	0.684	0.194
g08						309	364	344	339	23	243	181	258	227	33	0.788	0.529	0.659	0.130
g09						432	400	423	418	13	312	290	277	293	14	0.973	0.681	0.827	0.146
g10						314	308	214	279	46	97	128	133	119	16	0.648	0.278	0.463	0.185
regB	9	12	9	10	1														
g178	417	282	314	338	58														
g11						399	380	388	389	8	173	149	337	220	84	1.151	0.65	0.900	0.250
g13						504	625	501	543	58	205	314	242	254	45	1.607	0.75	1.179	0.429
g15						482	479	513	491	15	244	282	239	255	19	1.454	0.754	1.104	0.350
g17						382	328	396	369	29						1.091		0.645	0.223
regB	7	8	12	9	2														
g178	397	415	413	408	8														
g23						196	191	205	197	6						0.484		0.682	0.199
g48						620	680	666	655	26						1.606		1.711	0.105
g50						449	370	382	400	35						0.981		0.747	0.234
g51						242	279	239	253	18	100	125	94	106	13	0.621	0.261	0.441	0.180
regB	11	10	10	10	0														
g178	742	597	794	711	83														
g52						486	348	331	388	69	335	319	252	302	36	0.546	0.425	0.485	0.061

g64						322	348	231	300	50	216	133	170	173	34	0.422	0.243	0.333	0.090
g65						293	307	200	267	47						0.375		0.514	0.139
g68						362	282	485	376	83	236	275	286	266	21	0.529	0.374	0.451	0.078
regB	6	7	8	7	1														
g178	384	372	377	378	5														
g69						430	399	340	390	46	213	234	186	211	24	1.031	0.558	0.795	0.236
g76						750	601	693	681	75	297	275	326	299	26	1.802	0.792	1.297	0.505
g77						496	590	565	550	49	190	209	158	186	26	1.456	0.491	0.974	0.482
g23											308	315	342	322	18		0.881		
g50											226	161	175	187	34		0.513		
g65											206	245	265	239	30		0.654		
g17											216	255	235	235	20		0.645		
g48											650	660	679	663	15		1.816		
regB	14	7	14	12	3														
g178	381	314	399	365	37														

Table S10. NGS screening of phage fHy-Eco03 HPUF encoding genes. For each gene, two pairs of complementary sequences over the ligation joints were determined *in silico* containing 15-25 nt of the gene and plasmid specific sequences (the latter underlined). The joints named as *gx_atg* and *gx_stop* are sequences specific for the forward strand containing the start or stop codons of the inserted genes, while sequences named *gx_atgrev* and *gx_stoprev* are the corresponding complementary sequences from the reverse strand. Ligation Mix 1 and Mix 2 represent replicate ligation mixtures and Mix(1/2). Elp(1/2) represent replicate electroporations of the ligation mixes. The reads containing these joint sequences were extracted from the NGS sequence read files as described in Table S6. The joint-read totals for each gene and their percentages of total joint-reads were calculated. These were then used to calculate the plasmid DNA ligation mixture abundance ratios.

Gene	Joint	Joint sequence	Pooled ligations				Plasmid DNA from pooled transformants						% ratios
			Mix 1	Mix 2	*N* per gene	% per gene	Mix1Elp1	Mix1Elp2	Mix2Elp1	Mix2Elp2	*N* per gene	% per gene	
NGS experiment 1													
g01	*g01*_atg	CCAAGCTTGCATGCCTGCAGGCGGCCGCATGAGCTATACTGACCAACA	95	55			3562	3244	3162	2449			
	*g01*_atgrev	TGTTGGTCAGTATAGCTCATGCGGCCGCCTGCAGGCATGCAAGCTTGG	0	2			3930	3443	3243	2451			
	*g01*_stop	CCTGAGAGGTCAACTGTAACCATGGAGCTAGCTCTAGAGGATCCC	24	27			3704	3186	3293	2471			
	*g01*_stoprev	GGGATCCTCTAGAGCTAGCTCCATGGTTACAGTTGACCTCTCAGG	126	105	434	1,31	3665	3312	3257	2484	50856	3,52	2,69
g03	*g03*_atg	CCAAGCTTGCATGCCTGCAGGCGGCCGCGTGATTGGCTTAATCCTGG	104	81			5920	5084	4659	3780			
	*g03*_atgrev	CCAGGATTAAGCCAATCACGCGGCCGCCTGCAGGCATGCAAGCTTGG	2	2			5973	4799	4627	3435			
	*g03*_stop	CGATGAGTTGAAACAAGACTAACCATGGAGCTAGCTCTAGAGGATCCC	14	24			5938	5086	4863	3754			
	*g03*_stoprev	GGGATCCTCTAGAGCTAGCTCCATGGTTAGTCTTGTTTCAACTCATCG	103	88	418	1,26	5770	4927	4656	3613	76884	5,33	4,22
g04	*g04*_atg	CCAAGCTTGCATGCCTGCAGGCGGCCGCATGTTTAAATTCTTACAACGTAACC	58	53			3239	2652	2607	2197			
	*g04*_atgrev	GGTTACGTTGTAAGAATTTAAACATGCGGCCGCCTGCAGGCATGCAAGCTTGG	4	2			3014	2477	2352	1885			
	*g04*_stop	GTTGAAGGAGATTACTTGTGACCATGGAGCTAGCTCTAGAGGATCCC	26	18			3240	2553	2760	2184			
	*g04*_stoprev	GGGATCCTCTAGAGCTAGCTCCATGGTCACAAGTAATCTCCTTCAAC	77	56	294	0,89	3347	2763	2729	2243	42242	2,93	3,30
g05	*g05*_atg	CCAAGCTTGCATGCCTGCAGGCGGCCGCATGTTTTCTGAGGAGCAACT	283	178			0	3	4	0			
	*g05*_atgrev	AGTTGCTCCTCAGAAAACATGCGGCCGCCTGCAGGCATGCAAGCTTGG	2	1			0	0	0	0			
	*g05*_stop	ACTTGGAGTAAGGAGCAAATAACCATGGAGCTAGCTCTAGAGGATCCC	42	25			21	10	12	8			
	*g05*_stoprev	GGGATCCTCTAGAGCTAGCTCCATGGTTATTTGCTCCTTACTCCAAGT	335	248	1114	3,36	33	38	5	12	146	0,01	0,003
g06	*g06*_atg	CCAAGCTTGCATGCCTGCAGGCGGCCGCATGAAAAAGCATGTTGTTGAG	27	26			3666	3012	3342	2638			
	*g06*_atgrev	CTCAACAACATGCTTTTTCATGCGGCCGCCTGCAGGCATGCAAGCTTGG	8	11			3637	3076	3264	2626			
	*g06*_stop	GGTGTTGTCTTATGTTTTCTGACCATGGAGCTAGCTCTAGAGGATCCC	156	175			3611	3256	3578	2895			
	*g06*_stoprev	GGGATCCTCTAGAGCTAGCTCCATGGTCAGAAAACATAAGACAACACC	46	36	485	1,46	3562	3072	3373	2691	51299	3,56	2,43
g08	*g08*_atg	CCAAGCTTGCATGCCTGCAGGCGGCCGCATGCCATTAATCAAAGTTACAG	80	46			4148	3417	3805	3052			
	*g08*_atgrev	CTGTAACTTTGATTAATGGCATGCGGCCGCCTGCAGGCATGCAAGCTTGG	3	1			3528	3075	3626	2631			
	*g08*_stop	GTTACTGAGGTAATTATTTCTTGACCATGGAGCTAGCTCTAGAGGATCCC	41	26			4010	3509	4102	3220			
	*g08*_stoprev	GGGATCCTCTAGAGCTAGCTCCATGGTCAAGAAATAATTACCTCAGTAAC	121	106	424	1,28	3581	3219	3749	2867	55539	3,85	3,01
g09	*g09*_atg	CCAAGCTTGCATGCCTGCAGGCGGCCGCATGGTAACATTCACAACTATCC	251	142			5079	4545	3602	3039			
	*g09*_atgrev	GGATAGTTGTGAATGTTACCATGCGGCCGCCTGCAGGCATGCAAGCTTGG	10	3			5054	4295	3427	2766			
	*g09*_stop	GACGAATCAAAATTTGAGGTTTAACCATGGAGCTAGCTCTAGAGGATCCC	35	29			4920	4132	3430	2741			
	*g09*_stoprev	GGGATCCTCTAGAGCTAGCTCCATGGTTAAACCTCAAATTTTGATTCGTC	281	178	929	2,80	4617	4191	3383	2828	62049	4,30	1,53
g10	*g10*_atg	CCAAGCTTGCATGCCTGCAGGCGGCCGCATGATGAAAGAATTGACATTGAC	80	28			2089	1723	1148	916			
	*g10*_atgrev	GTCAATGTCAATTCTTTCATCATGCGGCCGCCTGCAGGCATGCAAGCTTGG	5	60			2152	1714	1290	943			
	*g10*_stop	TCAAATTAGTACAAAGTTACAATAGCCATGGAGCTAGCTCTAGAGGATCCC	23	11			2273	1595	1317	985			
	*g10*_stoprev	GGGATCCTCTAGAGCTAGCTCCATGGCTATTGTAACTTTGTACTAATTTGA	92	60	359	1,08	1807	1549	1120	846	23467	1,63	1,50
g11	*g11*_atg	CCAAGCTTGCATGCCTGCAGGCGGCCGCATGCATACTCAATTAATCATCTGG	71	67			2949	2514	2725	2207			

	*g11*_atgrev	CCAGATGATTAATTGAGTATGCATGCGGCCGCCTGCAGGCATGCAAGCTTGG	4	5			2945	2530	2821	2149			
	*g11*_stop	CACAATTTAACTCAGGAGAATGACCATGGAGCTAGCTCTAGAGGATCCC	29	20			3157	2815	3035	2447			
	*g11*_stoprev	GGGATCCTCTAGAGCTAGCTCCATGGTCATTCTCCTGAGTTAAATTGTG	90	68	354	1,07	3006	2491	2800	2282	42873	2,97	2,78
g13	*g13*_atg	CCAAGCTTGCATGCCTGCAGGCGGCCGCATGACTGTAGTTGCACCT	183	250			6229	5350	7414	5717			
	*g13*_atgrev	AGGTGCAACTACAGTCATGCGGCCGCCTGCAGGCATGCAAGCTTGG	2	7			4250	3905	5337	3939			
	*g13*_stop	TGGCTTAAATTTAAGGTAAAATGACCATGGAGCTAGCTCTAGAGGATCCC	12	21			4731	4310	6048	4987			
	*g13*_stoprev	GGGATCCTCTAGAGCTAGCTCCATGGTCATTTTACCTTAAATTTAAGCCA	224	251	950	2,87	4921	4266	6056	4915	82375	5,71	1,99
g15	*g15*_atg	CCAAGCTTGCATGCCTGCAGGCGGCCGCATGAACATCATAGTTGAGGGA	820	595			6502	5798	3773	3065			
	*g15*_atgrev	TCCCTCAACTATGATGTTCATGCGGCCGCCTGCAGGCATGCAAGCTTGG	3	5			6322	5494	3775	2777			
	*g15*_stop	CGATAAGGAGTGGACTAAATGACCATGGAGCTAGCTCTAGAGGATCCC	42	29			6070	5806	3783	3228			
	*g15*_stoprev	GGGATCCTCTAGAGCTAGCTCCATGGTCATTTAGTCCACTCCTTATCG	881	488	2863	8,64	6101	5753	3711	3146	75104	5,21	0,60
g17	*g17*_atg	CCAAGCTTGCATGCCTGCAGGCGGCCGCATGGACTTATTCGACTTAGTTGA	639	455			6207	5269	4906	4122			
	*g17*_atgrev	TCAACTAAGTCGAATAAGTCCATGCGGCCGCCTGCAGGCATGCAAGCTTGG	8	2			5034	4373	4366	3212			
	*g17*_stop	TGCTGGTAGAAATGGCATAACCATGGAGCTAGCTCTAGAGGATCCC	65	46			6295	5211	5472	4377			
	*g17*_stoprev	GGGATCCTCTAGAGCTAGCTCCATGGTTATGCCATTTCTACCAGCA	809	555	2579	7,79	5995	5116	5124	4126	79205	5,49	0,71
g23	*g23*_atg	CCAAGCTTGCATGCCTGCAGGCGGCCGCATGGGTGGCGCAAGAATC	689	461			3220	2952	2936	2134			
	*g23*_atgrev	GATTCTTGCGCCACCCATGCGGCCGCCTGCAGGCATGCAAGCTTGG	4	4			3019	2658	2716	2228			
	*g23*_stop	TTTACTTGTACAAAATTAGCCATGGAGCTAGCTCTAGAG	68	44			3380	3204	3293	2587			
	*g23*_stoprev	CTCTAGAGCTAGCTCCATGGCTAATTTTGTACAAGTAAA	1522	1213	4005	12,09	3655	3378	3346	2680	47386	3,28	0,27
g48	*g48*_atg	CCAAGCTTGCATGCCTGCAGGCGGCCGCTTGACCTCATGCAGTAAAAAG	241	182			16372	14621	14149	11134			
	*g48*_atgrev	CTTTTTACTGCATGAGGTCAAGCGGCCGCCTGCAGGCATGCAAGCTTGG	6	3			14949	13359	12842	9941			
	*g48*_stop	AGTGAAGGGGCCAATTAACCATGGAGCTAGCTCTAGAGGATCCC	87	79			15501	14418	14516	11213			
	*g48*_stoprev	GGGATCCTCTAGAGCTAGCTCCATGGTTAATTGGCCCCTTCACT	282	247	1127	3,40	16598	15055	15410	11945	222023	15,39	4,52
g50	*g50*_atg	CCAAGCTTGCATGCCTGCAGGCGGCCGCATGATTGAATCTACACGCAC	244	162			5148	4293	3726	2957			
	*g50*_atgrev	GTGCGTGTAGATTCAATCATGCGGCCGCCTGCAGGCATGCAAGCTTGG	5	3			4566	3610	2966	2280			
	*g50*_stop	AAAAGAAAGGAAAGCGCTAACCATGGAGCTAGCTCTAGAGGATCCC	40	41			4639	3793	3206	2737			
	*g50*_stoprev	GGGATCCTCTAGAGCTAGCTCCATGGTTAGCGCTTTCCTTTCTTTT	233	155	883	2,67	4692	3994	3447	2807	58861	4,08	1,53
g51	*g51*_atg	CCAAGCTTGCATGCCTGCAGGCGGCCGCATGAGAAAGTTTACCCTGGA	50	42			5333	4457	4895	3957			
	*g51*_atgrev	TCCAGGGTAAACTTTCTCATGCGGCCGCCTGCAGGCATGCAAGCTTGG	4	4			6204	5176	5605	4309			
	*g51*_stop	ACTTGGCTAAGAAATATGATTGACCATGGAGCTAGCTCTAGAGGATCCC	27	9			5561	4843	5635	4481			
	*g51*_stoprev	GGGATCCTCTAGAGCTAGCTCCATGGTCAATCATATTTCTTAGCCAAGT	85	80	301	0,91	5020	4254	4697	3650	78077	5,41	5,96
g52	*g52*_atg	CCAAGCTTGCATGCCTGCAGGCGGCCGCGTGTCTAATATAGGTTTAGAGTACC	562	502			4254	3525	3656	2843			
	*g52*_atgrev	GGTACTCTAAACCTATATTAGACACGCGGCCGCCTGCAGGCATGCAAGCTTGG	6	19			3384	2739	3070	2244			
	*g52*_stop	CGAGGAAGAATGCAAATGACCATGGAGCTAGCTCTAGAGGATCCC	35	40			4448	3531	3906	3292			
	*g52*_stoprev	GGGATCCTCTAGAGCTAGCTCCATGGTCATTTGCATTCTTCCTCG	606	465	2235	6,75	4127	3439	3598	2899	54955	3,81	0,56
g64	*g64*_atg	CCAAGCTTGCATGCCTGCAGGCGGCCGCATGTTTGTGCCGATTCCG	837	497			2055	2072	1918	1371			
	*g64*_atgrev	CGGAATCGGCACAAACATGCGGCCGCCTGCAGGCATGCAAGCTTGG	9	4			1933	1847	1814	1396			
	*g64*_stop	CGCTGAAGAAGCGAATTGACCATGGAGCTAGCTCTAGAGGATCCC	30	17			2068	2012	1976	1520			
	*g64*_stoprev	GGGATCCTCTAGAGCTAGCTCCATGGTCAATTCGCTTCTTCAGCG	746	479	2619	7,91	2320	1994	2049	1575	29920	2,07	0,26
g65	*g65*_atg	CCAAGCTTGCATGCCTGCAGGCGGCCGCATGCAGAACCTTTGGGATA	533	444			5013	4535	5843	4669			
	*g65*_atgrev	TATCCCAAAGGTTCTGCATGCGGCCGCCTGCAGGCATGCAAGCTTGG	0	3			4243	3836	5056	3961			
	*g65*_stop	CCGGCTACACAAGCTGACCATGGAGCTAGCTCTAGAGGATCCC	27	30			4708	4347	5995	4460			
	*g65*_stoprev	GGGATCCTCTAGAGCTAGCTCCATGGTCAGCTTGTGTAGCCGG	380	369	1786	5,39	5268	4669	6475	4800	77878	5,40	1,00
g68	*g68*_atg	CCAAGCTTGCATGCCTGCAGGCGGCCGCATGTATACGTTCAATCAATTCAAA	293	205			1348	1086	846	678			
	*g68*_atgrev	TTTGAATTGATTGAACGTATACATGCGGCCGCCTGCAGGCATGCAAGCTTGG	8	7			1157	1034	757	553			
	*g68*_stop	GATGTTAACAGAGGGGTATTAACCATGGAGCTAGCTCTAGAGGATCCC	42	42			1313	1020	936	678			
	*g68*_stoprev	GGGATCCTCTAGAGCTAGCTCCATGGTTAATACCCCTCTGTTAACATC	336	229	1162	1,31	1305	1039	848	732	15330	1,06	0,81
g69	*g69*_atg	CCAAGCTTGCATGCCTGCAGGCGGCCGCATGGAAGCCACCAAGTCT	965	556			6676	5661	5057	4117			

	*g69*_atgrev	AGACTTGGTGGCTTCCATGCGGCCGCCTGCAGGCATGCAAGCTTGG	3	2			6311	5534	4816	3859			
	*g69*_stop	GTGTACCATTGAAGTGTATAAATAACCATGGAGCTAGCTCTAGAGGATCCC	19	11			5559	4802	4518	3431			
	*g69*_stoprev	GGGATCCTCTAGAGCTAGCTCCATGGTTATTTATACACTTCAATGGTACAC	687	467	2710	8,18	6244	5494	5048	4024	81151	5,62	0,69
g76	*g76*_atg	AGCTTGCATGCCTGCAGGCGGCCGCATGAAACATCAGGGC	355	302			5815	4711	4355	3411			
	*g76*_atgrev	GCCCTGATGTTTCATGCGGCCGCCTGCAGGCATGCAAGCT	1	2			4961	4305	3949	3037			
	*g76*_stop	CATGCACGCTGGAGATAACCATGGAGCTAGCTCTAGAGGATCCC	40	26			4816	3867	3986	2842			
	*g76*_stoprev	GGGATCCTCTAGAGCTAGCTCCATGGTTATCTCCAGCGTGCATG	353	226	1305	3,94	5026	4241	3925	3084	66331	4,60	1,17
g77	*g77*_atg	CCAAGCTTGCATGCCTGCAGGCGGCCGCATGAAAACAATGTTTGTATACAAG	1426	870			5061	3957	5141	4112			
	*g77*_atgrev	CTTGTATACAAACATTGTTTTCATGCGGCCGCCTGCAGGCATGCAAGCTTGG	12	6			3902	3272	4273	3045			
	*g77*_stop	GAGGTGTTAGGCCTGTGACCATGGAGCTAGCTCTAGAGGATCCC	49	35			4599	3988	5007	4006			
	*g77*_stoprev	GGGATCCTCTAGAGCTAGCTCCATGGTCACAGGCCTAACACCTC	709	681	3788	11,44	4720	4073	5396	4310	68862	4,77	0,42
		**Totals**			**33124**						**1442813**		
NGS experiment 2													
g18	*g18*_atg	CCAAGCTTGCATGCCTGCAGGCGGCCGCATGGCACGAAAACCACAA	594	568			32026	25686	27758	26039			
	*g18*_atgrev	TTGTGGTTTTCGTGCCATGCGGCCGCCTGCAGGCATGCAAGCTTGG	4	0			24355	19722	20933	19404			
	*g18*_stop	GGACGAGGATAATAATGAATCTTGACCATGGAGCTAGCTCTAGAGGATCCC	16	4			23707	18651	20102	18915			
	*g18*_stoprev	GGGATCCTCTAGAGCTAGCTCCATGGTCAAGATTCATTATTATCCTCGTCC	415	291	1892	36,90	24199	18668	19867	19246	359278	41,23	1,12
g26	*g26*_atg	CCAAGCTTGCATGCCTGCAGGCGGCCGCATGGGCCTGTTGAAGATTAC	215	206			8285	6530	7396	7384			
	*g26*_atgrev	GTAATCTTCAACAGGCCCATGCGGCCGCCTGCAGGCATGCAAGCTTGG	2	0			7618	6473	7369	6877			
	*g26*_stop	CCGGCTTTTACAATTACCTGACCATGGAGCTAGCTCTAGAGGATCCC	19	18			7373	5499	6399	5986			
	*g26*_stoprev	GGGATCCTCTAGAGCTAGCTCCATGGTCAGGTAATTGTAAAAGCCGG	295	231	986	19,23	7943	6355	7247	6830	111564	12,80	0,67
g40	*g40*_atg	CCAAGCTTGCATGCCTGCAGGCGGCCGCATGAGAACCAGACGACTTGA	99	58			2351	2162	2007	1861			
	*g40*_atgrev	TCAAGTCGTCTGGTTCTCATGCGGCCGCCTGCAGGCATGCAAGCTTGG	1	2			1804	1743	1680	1500			
	*g40*_stop	GACACTGTTGCGGGGTAGCCATGGAGCTAGCTCTAGAGGATCCC	10	18			2413	1935	1954	1679			
	*g40*_stoprev	GGGATCCTCTAGAGCTAGCTCCATGGCTACCCCGCAACAGTGTC	258	142	588	11,47	2582	2104	2045	1833	31653	3,63	0,32
g58	*g58*_atg	CCAAGCTTGCATGCCTGCAGGCGGCCGCTTGGATATTCATGCTTCGCTC	0	0			190	96	141	104			
	*g58*_atgrev	GAGCGAAGCATGAATATCCAAGCGGCCGCCTGCAGGCATGCAAGCTTGG	0	0			188	125	171	121			
	*g58*_stop	AACAAGGATGGTGTTGCATGACCATGGAGCTAGCTCTAGAGGATCCC	7	10			268	172	240	252			
	*g58*_stoprev	GGGATCCTCTAGAGCTAGCTCCATGGTCATGCAACACCATCCTTGTT	23	9	49	0,96	249	153	190	191	2851	0,33	0,34
g59	*g59*_atg	CCAAGCTTGCATGCCTGCAGGCGGCCGCTTGAATGTAGCATTAAGTGTGGC	6	4			11217	8583	9104	8817			
	*g59*_atgrev	GCCACACTTAATGCTACATTCAAGCGGCCGCCTGCAGGCATGCAAGCTTGG	0	0			10005	8543	8755	8422			
	*g59*_stop	GTTCGAGTCTGCTCGCTGACCATGGAGCTAGCTCTAGAGGATCCC	13	10			11608	8909	9883	9073			
	*g59*_stoprev	GGGATCCTCTAGAGCTAGCTCCATGGTCAGCGAGCAGACTCGAAC	33	55	121	2,36	11051	8558	9485	8459	150472	17,27	7,32
g61	*g61*_atg	CCAAGCTTGCATGCCTGCAGGCGGCCGCTTGGGCAACCGAGAACAG	30	29			7293	6429	4773	4955			
	*g61*_atgrev	CTGTTCTCGGTTGCCCAAGCGGCCGCCTGCAGGCATGCAAGCTTGG	0	0			6223	5908	4512	4592			
	*g61*_stop	CTTTTGACTATAAAGTGCCTTTTTAGCCATGGAGCTAGCTCTAGAGGATCCC	10	1			5433	4700	3700	3641			
	*g61*_stoprev	GGGATCCTCTAGAGCTAGCTCCATGGCTAAAAAGGCACTTTATAGTCAAAAG	44	25	139	2,71	6233	5290	4055	4224	81961	9,41	3,47
g71	*g71*_atg	CCAAGCTTGCATGCCTGCAGGCGGCCGCATGAGTGAGAACTTCGAGAAG	124	125			6723	5962	5272	5492			
	*g71*_atgrev	CTTCTCGAAGTTCTCACTCATGCGGCCGCCTGCAGGCATGCAAGCTTGG	0	1			6472	6138	5315	5338			
	*g71*_stop	TGCGAAGGGGCACTAAAATAACCATGGAGCTAGCTCTAGAGGATCCC	19	13			6720	5587	4838	5011			
	*g71*_stoprev	GGGATCCTCTAGAGCTAGCTCCATGGTTATTTTAGTGCCCCTTCGCA	182	130	594	11,59	6759	6080	5171	5267	92145	10,57	0,91
g72	*g72*_atg	CCAAGCTTGCATGCCTGCAGGCGGCCGCATGAAAGCGGTCAACAAGAA	17	25			1487	1452	1262	1081			
	*g72*_atgrev	TTCTTGTTGACCGCTTTCATGCGGCCGCCTGCAGGCATGCAAGCTTGG	0	2			1321	1267	1109	977			
	*g72*_stop	GTACAGGTAGAACTGCCATGACCATGGAGCTAGCTCTAGAGGATCCC	11	11			1585	1617	1191	1126			
	*g72*_stoprev	GGGATCCTCTAGAGCTAGCTCCATGGTCATGGCAGTTCTACCTGTAC	85	58	209	4,08	1620	1506	1320	1212	21133	2,43	0,59
g73	*g73*_atg	CCAAGCTTGCATGCCTGCAGGCGGCCGCATGGCGACGAGTAAAAACG	51	42			1686	1305	1175	1238			
	*g73*_atgrev	CGTTTTTACTCGTCGCCATGCGGCCGCCTGCAGGCATGCAAGCTTGG	0	0			1444	1190	1097	1058			

*g73*_stop	CAAGAAGTACAGTCAGGAAGATAACCATGGAGCTAGCTCTAGAGGATCCC	22	17			1464	1224	1145	1083			
*g73*_stoprev	GGGATCCTCTAGAGCTAGCTCCATGGTTATCTTCCTGACTGTACTTCTTG	268	149	549	10,71	1612	1294	1187	1196	20398	2,34	0,22
		**Totals**		**5127**						**871455**		

References

1. Mohanraj, U.; Wan, X.; Spruit, C.M.; Skurnik, M.; Pajunen, M.I. A toxicity screening approach to identify bacteriophage-encoded anti-microbial proteins. *Viruses* **2019**, *11*.
2. Zimmermann, L.; Stephens, A.; Nam, S.Z.; Rau, D.; Kubler, J.; Lozajic, M.; Gabler, F.; Soding, J.; Lupas, A.N.; Alva, V. A Completely Reimplemented MPI Bioinformatics Toolkit with a New HHpred Server at its Core. *J Mol Biol* **2018**, *430*, 2237-2243.
